# The Impact of Behavioural Economics‐Informed Interventions on the Care Cascade for Sexually Transmitted and Blood‐Borne Infections: A Systematic Review of Randomized Controlled Trials

**DOI:** 10.1002/jia2.70136

**Published:** 2026-08-10

**Authors:** Min Zhao, Zhengcheng Tu, Xiang Zhao, Chunyan Li, Jiajun Sun, Ye Zhang, Warittha Tieosapjaroen, Fanpu Ji, Jason J. Ong, Lei Zhang

**Affiliations:** ^1^ Phase I clinical trial research ward The Second Affiliated Hospital of Xi'an Jiaotong University Xi'an People's Republic of China; ^2^ China‐Australia Joint Research Centre for Infectious Diseases School of Public Health Xi'an Jiaotong University Xi'an People's Republic of China; ^3^ School of Translational Medicine Monash University Melbourne Victoria Australia; ^4^ Second Department of Coronary Heart Disease The First Affiliated Hospital of Xinjiang Medical University Urumqi People's Republic of China; ^5^ Tokyo College The University of Tokyo Tokyo Japan; ^6^ School of Public Policy and Administration Xi'an Jiaotong University Xi'an People's Republic of China; ^7^ Melbourne Sexual Health Centre Alfred Health Melbourne Victoria Australia; ^8^ School of Public Health Nanjing Medical University Nanjing People's Republic of China; ^9^ Kirby Institute UNSW Sydney Sydney New South Wales Australia

**Keywords:** behavioural economics, HIV, incentives, nudge, pay‐it‐forward, sexually transmitted and blood‐borne infections

## Abstract

**Introduction:**

While behavioural economics principles are widely used for public health interventions, a comprehensive understanding of their impact across the sexually transmitted and blood‐borne infections (STBBIs) care cascade remains limited. We systematically evaluated the effectiveness of behavioural economics‐informed interventions for an STBBI care cascade.

**Methods:**

We systematically searched five major literature databases from their inception to 25 April 2024, with the search updated on 22 March 2025. We included randomized controlled trials (RCTs) evaluating behavioural economics‐informed interventions, that is strategies such as incentives, defaults, framing and reminders designed to influence health‐related decision‐making, at any stage of the care cascade for STBBIs. The cascade included three domains: prevention, care engagement and treatment adherence. Two reviewers independently extracted data and assessed the risk of bias using the Cochrane RoB‐2 tool. Due to significant heterogeneity, findings were synthesized narratively using a three‐stage care cascade framework.

**Results:**

Our search identified 2546 records, of which 40 RCTs met the inclusion criteria and evaluated 12 distinct behavioural economics‐informed intervention types. Incentives were the most common intervention, including monetary, lottery and voucher‐based incentives (25/40; 63%). Monetary and voucher incentives effectively improved outcomes across multiple cascade stages, including prevention (such as safer sex, male medical circumcision), care engagement (testing for HIV, HBV and HCV) and treatment adherence (such as retention in care, viral suppression). Opt‐out defaults showed strong, sustained effects on HIV/HCV screening. Pay‐it‐forward strategies significantly increased dual STBBI testing and often demonstrated cost‐effectiveness. A substantial proportion of studies (27/40; 67.5%) reported a high risk of bias.

**Discussion:**

Behavioural economics‐informed interventions showed stage‐specific effects across the STBBI care cascade. Monetary incentives were the most consistently effective, especially for prevention, testing and some adherence outcomes. Defaults and pay‐it‐forward were promising for screening uptake, whereas crowdsourcing appeared more useful for intervention design. Overall, effectiveness depended on intervention design and context.

**Conclusions:**

Behavioural economics‐informed interventions hold substantial promise for optimizing the STBBI care cascade, but no single strategy was universally optimal. Future research should strengthen the evidence base and assess the transferability of these interventions across diseases and context‐specific settings to inform wider implementation and maximize global public health impact.

## 1 Introduction

Sexually transmitted and blood‐borne infections (STBBIs), including viral infections and bacterial infections, pose a significant global health challenge. Each day, over one million individuals acquire curable sexually transmitted infections (STIs), a burden that includes bacterial infections such as chlamydia, gonorrhoea and syphilis, while viral infections like human immunodeficiency virus (HIV), human papillomavirus (HPV), herpes simplex virus (HSV), hepatitis B virus (HBV) and hepatitis C virus (HCV) add further complexity to the global disease burden through their chronicity and potential for long‐term complications [1]. Despite notable advances in prevention, diagnosis and treatment, STBBIs, particularly HIV, continue to cause substantial morbidity and mortality, with significant disparities in care and outcomes, especially in low‐ and middle‐income countries (LMICs) [2]. Effective navigation of the STBBI care continuum, spanning prevention, care engagement and treatment adherence, necessitates multifaceted interventions to mitigate barriers that impede timely and effective care [3, 4, 5]. Although biomedical interventions are foundational to STBBI management, the ultimate success of prevention and care efforts is critically dependent on addressing social, psychological and behavioural determinants, such as individual motivation, stigma and health literacy [6, 7, 8]. In this context, behavioural economics‐informed interventions, which leverage insights from psychology and economics, emerge as a promising modality for targeting these non‐clinical barriers and improving health outcomes across the care cascade [9, 10].

The discipline of behavioural economics integrates behavioural psychology with traditional economic theory, challenging the assumption of fully rational decision‐making by highlighting cognitive biases, time preferences and social pressures that influence individual behaviour [11, 12]. Conventional interventions often overlook these non‐rational factors, which can hinder efforts to encourage behaviours, such as regular STBBI screening, timely treatment initiation and adherence to care regimens [13]. By focusing on how individuals perceive and respond to risks, rewards and social norms, interventions informed by behavioural economics seek to “nudge” individuals, that is to subtly guide them towards “healthier” decisions without restricting their freedom of choice [13]. Examples of behavioural economics‐informed interventions include incentive‐based strategies, default options, framed messages, reminders or planning prompts and socially oriented approaches such as pay‐it‐forward and crowdsourcing, which can be used to promote condom use, encourage regular testing or support adherence to treatment regimens [14, 15, 16]. These approaches often involve low‐cost, easy‐to‐implement change techniques that can be integrated into existing health systems, potentially leading to significant improvements in STBBI care uptake and outcomes [15].

Previous reviews have explored how behavioural economics‐informed strategies were used for HIV prevention and management. For example, Long and Devine conducted a scoping review that applied the EAST (Easy, Attractive, Social, Timely) framework to categorize behavioural economics‐informed interventions in HIV programmes within LMICs [16]. Their work focused on simplifying processes, increasing the salience of interventions and encouraging timely behaviours to improve HIV outcomes. Similarly, Andrawis et al. utilized the MINDSPACE framework (Messenger, Incentives, Norms, Defaults, Salience, Priming, Affect, Commitments and Ego) to synthesize behavioural insights applied to HIV prevention and management [14]. This review highlighted the range of “nudges” used to influence health behaviours and decisions in HIV care. Building on these foundational studies, Ahmed and McNamee conducted a systematic review of randomized controlled trials (RCTs) that assessed the effectiveness of behavioural economics‐informed interventions for HIV prevention, screening and antiretroviral treatment, delivering a comprehensive analysis of behavioural economics‐informed strategies in their application to HIV care [15].

While existing reviews provide critical insights into behavioural economics‐informed strategies for HIV management [14, 15, 16], focusing on a single disease overlooks the principle that interventions targeting universal cognitive biases may have transferable utility across diverse STBBI contexts. Although STBBIs differ in natural history, stigma and care pathways, many intervention targets remain concentrated in key behavioural stages, including prevention, testing uptake, treatment initiation and sustained adherence [17, 18, 19]. Across different stages of the care cascade, individuals may face similar behavioural frictions, such as present bias, optimism bias, procrastination, hassle costs and responsiveness to defaults or social reciprocity [20, 21]. Behavioural economics‐informed interventions are often designed to address these frictions [22, 23]. For this reason, examining interventions across STBBIs may help identify shared behavioural mechanisms and cross‐disease patterns in intervention applicability that may be less visible in disease‐specific reviews. However, systematic evidence on this issue remains limited, and it is not yet clear which strategies show cross‐disease relevance, where evidence is concentrated, and which diseases or cascade stages remain insufficiently studied.

To address this gap, this review systematically evaluates the effectiveness of behavioural economics‐informed interventions across the STBBI care cascade based on RCTs. By mapping STBBI care‐related outcomes to prevention, care engagement and treatment adherence stages, this study synthesizes evidence on behavioural economic strategies demonstrating efficacy in modulating these behaviours. In doing so, this review will contribute to the growing body of literature on behavioural economics in health interventions, offering actionable insights for behavioural interventionists, policymakers, healthcare providers and researchers working to enhance STBBI prevention and care.

## 2 Methods

The review adhered to the PRISMA (Preferred Reporting Items for Systematic Reviews and Meta‐Analyses) 2020 guidelines [24], and the protocol was registered on PROSPERO (CRD42024541068).

### 2.1 Search Strategy and Selection Criteria

A comprehensive search was conducted across five major databases: MEDLINE (via PubMed), Embase, CINAHL (via EBSCOhost), Web of Science (Core Collection) and the Cochrane Central Register of Controlled Trials (CENTRAL). The literature search was conducted from database inception to 25 April 2024 and updated on 22 March 2025. No restrictions were placed on language, publication date or geographical setting. The studies ultimately included in this review were published between 2012 and 2024. The review was completed in August 2025.

The search strategy combined controlled vocabulary (e.g. MeSH terms) and free‐text keywords to identify relevant studies. Search terms related to STBBI, including both viral infections (HIV, HPV, HSV, HBV, HCV) and bacterial infections (syphilis, gonorrhoea, chlamydia), were combined with terms for behavioural economics‐informed strategies. For this review, behavioural economics‐informed strategies were defined as interventions that apply principles or theory from behavioural economics to guide individuals towards healthier choices [11]. These included general terms like “nudg*,” “behavi* economics” and “behavi* intervention,” as well as keywords for specific behavioural economics concepts such as “choice architecture,” “pay‐it‐forward.” Additionally, to capture studies that used participatory methods for developing behavioural economics‐informed interventions, we included terms like “crowdsourcing” and “nudgeathon” [25, 26]. These terms were applied across titles, abstracts, keywords and full texts. Search filters specific to RCTs were employed where applicable to align with the eligibility criteria. The full search strategies for each database are detailed in Appendix S1. In addition to database searches, the reference lists of all included studies and relevant systematic reviews were manually screened to identify any additional studies.

**FIGURE 1:**
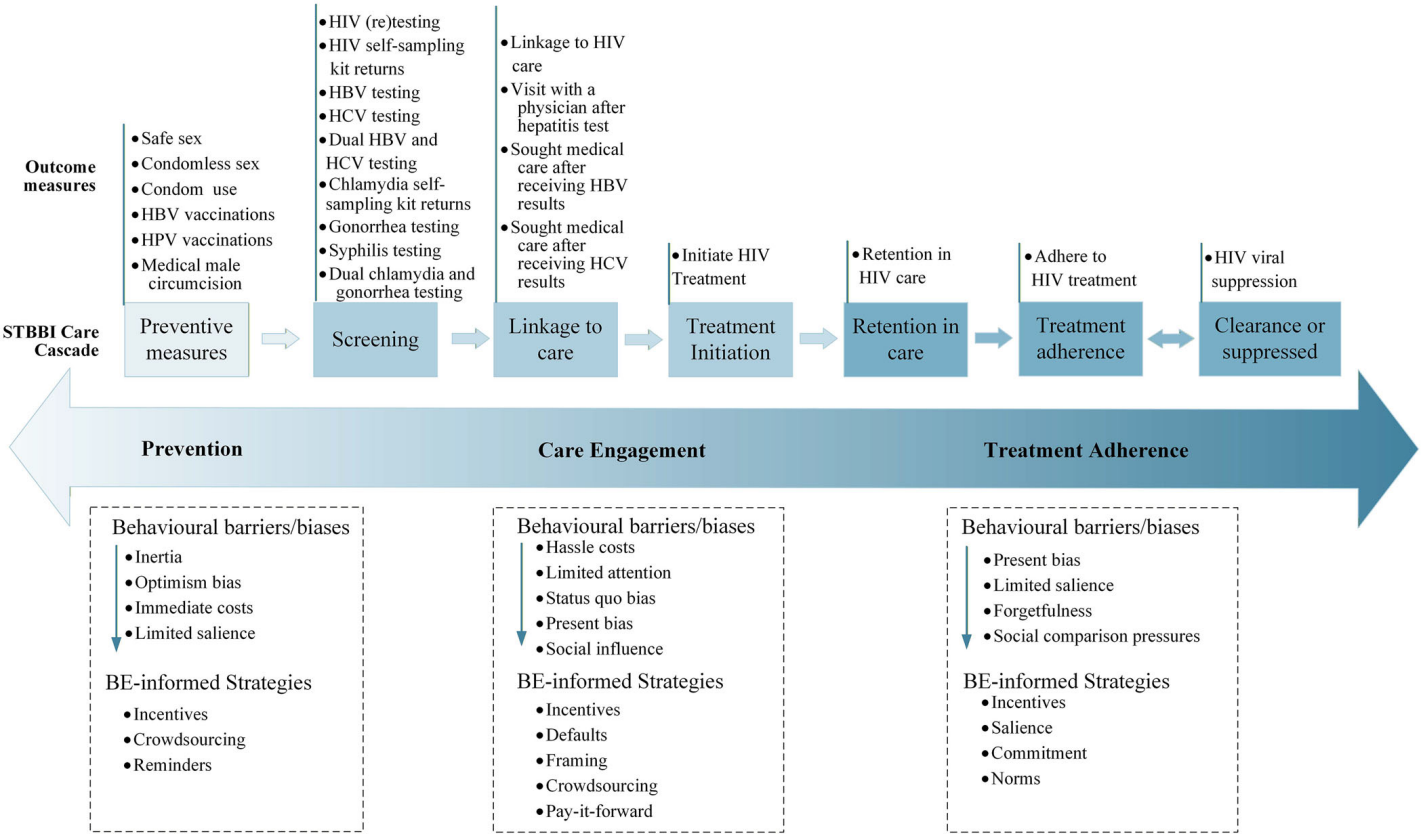
Adapted STBBI care cascade framework showing outcome domains, key behavioural barriers/biases and behavioural economics‐informed strategies. Abbreviations: BE‐informed, behavioural economics‐informed; HBV, hepatitis B virus; HCV, hepatitis C virus; HIV, human immunodeficiency virus; STBBI, sexually transmitted and blood‐borne infections.

Inclusion criteria were established using the PICO framework (Population, Intervention, Comparison, Outcome). For Population (P), studies targeting any population at risk for or affected by STBBIs were included. For Intervention (I), studies were included if they explicitly applied recognized behavioural economics concepts, such as interventions labelled as “nudges” or if the authors framed their intervention as being behavioural economics‐informed. For Comparison (C), all types of control groups were considered. For Outcome (O), studies were eligible if they reported at least one STBBI care cascade‐related primary or secondary outcome (Figure 1). Additionally, all studies had to employ an RCT design and be published in English in peer‐reviewed journals.

Studies were excluded if they met any of the following criteria: (1) irrelevance to interventions utilizing behavioural economics‐informed strategies; (2) inclusion of reviews, meta‐analyses, observational studies, study protocols, editorials, letters, comments or case reports; (3) duplicate publications reporting on previously included trials, including secondary analyses, follow‐up studies and subgroup analyses, were not included as separate studies to avoid data duplication; (4) availability as abstracts only, lack of retrievable full text or no reporting of STBBI care cascade‐related outcomes; (5) publication in a language other than English; or (6) being outside the scope of this review as judged by two independent researchers (MZ and ZT).

### 2.2 Data Collection and Definition

All records retrieved from the searches were imported into the Rayyan software for de‐duplication and screening. Titles, abstracts and keywords were independently screened by two reviewers (MZ and ZT) to identify potentially eligible studies. Full‐text articles of studies that met the inclusion criteria were retrieved and reviewed in detail. Disagreements during the screening process were resolved through discussion or, when necessary, by a third reviewer (LZ). In this study, a total of 25 conflicting articles were adjudicated by the third independent reviewer to determine their inclusion or exclusion. The inclusion of studies was finalized only when a consensus was reached among reviewers. The study selection process, including reasons for exclusions at the full‐text stage, is presented in a PRISMA flow diagram.

Data extraction was performed independently by two reviewers using the Systematic Review Data Repository Plus (SRDR+), an online collaborative tool for systematic reviews. Extracted data included study characteristics (e.g. authors, publication year, study setting and sample size), participant demographics, behavioural economics‐informed intervention descriptions, comparators and reported outcomes. Effect measures, including adjusted relative risk (ARR), odds ratio (OR), adjusted difference (AD), risk difference (RD), mean differences and corresponding 95% confidence intervals (CIs), were extracted for quantitative synthesis. For studies with missing or unclear data, attempts were made to contact the study authors for clarification.

For clarity, behavioural economics‐informed interventions in this review were grouped into several broad categories. Incentive‐based strategies included monetary, voucher and lottery incentives, for example cash rewards for HIV testing or vouchers for male medical circumcision (MMC) [27, 28, 29]. Choice architecture and message‐based strategies included defaults (e.g. opt‐out HIV testing) [29, 30], framing (e.g. gain‐framed testing messages) [31, 32], commitment approaches (e.g. deposit contracts) [33, 34], norm‐based interventions (e.g. messages highlighting peer participation) [30], planning prompts and reminders (e.g. prompts to return self‐testing kits or reminder messages) [35, 36] and salience‐enhancing strategies (e.g. messages drawing attention to the benefits of screening) [37]. Socially oriented approaches included pay‐it‐forward models (e.g. receiving a free test supported by previous participants and contributing to the next person) [38, 39] and crowdsourcing (e.g. community‐generated materials to promote testing) [40]. A fuller description of these intervention domains, along with illustrative examples, is provided in Table S1 (Appendix S4).

The outcomes in this review were mapped to a unified STBBI care cascade framework (Figure 1), adapted from established conceptual models [3, 4, 5]. The cascade was divided into three domains: prevention, care engagement and treatment adherence. Prevention includes risk reduction and vaccination; care engagement includes screening, linkage to care and treatment initiation; and treatment adherence includes retention in care, treatment adherence and clearance or viral suppression. In this adapted framework, each cascade stage is linked to the key behavioural barriers or biases and the corresponding behavioural economics‐informed strategies identified from the included RCTs. This integrated framework provides a structured basis for classifying outcomes from the included studies and for interpreting how behavioural economics‐informed interventions may influence progression across the STBBI care cascade.

### 2.3 Analysis

A narrative synthesis of the findings was conducted due to significant heterogeneity in intervention types, study populations and outcome measures. Specifically, the included studies examined a variety of STBBI and diverse stages of the STBBI care cascade, employing a wide range of behavioural economics‐informed interventions. The core principles and the specific application of behavioural economics‐informed interventions in each RCT are detailed in Table S1. This diversity in outcomes and interventions, alongside variations in study populations, precluded a meta‐analysis. Studies were grouped according to the stages of the STBBI care cascade they targeted and the behavioural economics‐informed strategies employed. Results were presented in structured tables and figures, summarizing study characteristics, comparisons, intervention outcomes and test statistics. Potential reporting bias was qualitatively assessed by examining selective outcome reporting. The overall quality of evidence was described narratively, contextualizing the findings, although no formal grading was performed.

The risk of bias for each included study was assessed independently by two reviewers using the Cochrane Risk of Bias Tool 2 (RoB‐2). The 2019 version of RoB‐2 was applied to individual RCTs, while the 2021 version was used for cluster RCTs [41]. Thirty‐four studies were assessed using an Excel tool to implement RoB‐2, and six studies were assessed using an Excel tool to implement cluster RoB‐2. Each study received an overall risk‐of‐bias judgement categorized as low risk, some concerns or high risk. Any discrepancies between reviewers were resolved through consensus. In addition, to provide study‐level context for interpreting the robustness of the review findings, Table S2 (Appendix S4) summarizes the direction of effect, overall RoB judgement, sample size and follow‐up duration for each included RCT.

### 2.4 Ethical Considerations

Ethical approval and informed consent were not required for this systematic review as it was based on previously published studies, and no primary data were collected from human or animal subjects.

### 2.5 Role of the Funding Source

The funding sources had no role in the study design, data collection, analysis, interpretation, writing of the paper or decision to submit the paper for publication.

## 3 Results

### 3.1 Study Selection

Our search identified a total of 2546 records. The initial search of five major databases yielded 2461 records. After removing 1042 duplicate records, 1419 records were screened. Among these, 1029 records were excluded as irrelevant, 12 as duplicates, 317 as non‐RCTs and 32 as conference abstracts with no detailed results, resulting in 29 eligible records. An additional 10 records were identified through citation searching. A second‐round search conducted before full‐text analysis yielded 75 records from targeted database alerts and identified one additional eligible study. In total, 40 studies were included in the final review. The study selection process is summarized in the PRISMA flow diagram (Appendix S3 and Figure S1).

### 3.2 Study Characteristics of Included RCTs

**FIGURE 2:**
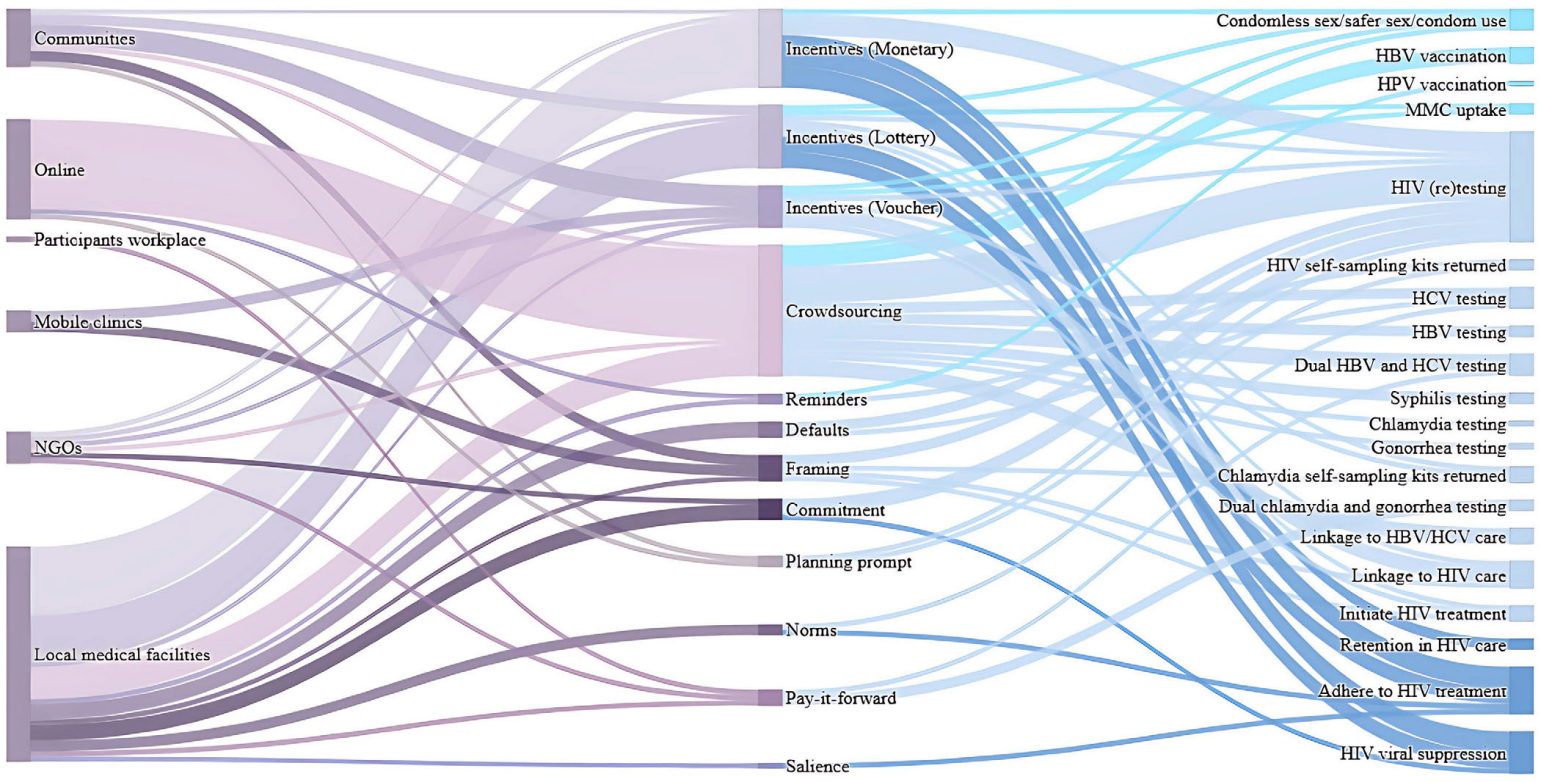
Relationships between implementation settings (left), behavioural economics‐informed interventions (middle) and STBBI outcomes (right) of 40 RCTs. The bar widths indicate the frequencies of each item. Abbreviations: HBV, hepatitis B virus; HCV, hepatitis C virus; HIV, human immunodeficiency virus; MMC, medical male circumcision; STBBI, sexually transmitted and blood‐borne infections.

The included RCTs utilized a range of behavioural economics‐informed strategies (Appendix S2). Figure 2 demonstrates extensive utilization of incentive‐based strategies (monetary, lottery, voucher), frequently directed towards STBBI prevention outcomes (e.g. MMC, safer sex) and HIV (re)testing. Concurrently, default strategies were commonly applied to HIV/HCV testing. Crowdsourcing interventions were often linked to HIV (re)testing and hepatitis testing. “Pay‐it‐forward” principles were frequently linked to dual STBBI testing outcomes. Most studies were conducted in Africa (*n* = 21), with others in China (*n* = 9), the United States (*n* = 6), England (*n* = 2) and Ecuador (*n* = 2) (Appendix S3 and Figure S2). Overall, the evidence base was unevenly distributed across diseases and care cascade stages, with 29 of the 40 included RCTs (72.5%) reporting at least one HIV‐related outcome.

### 3.3 Impact of Behavioural Economics‐Informed Interventions on STBBI Prevention

Behavioural economics‐informed interventions have shown significant promise in promoting STBBI prevention (Table 1). For instance, one study demonstrated a significant reduction in the prevalence of four STIs at 12 months among young adults in rural Tanzania receiving USD 20 monetary incentives for engaging in safe sex behaviours, compared to standard of care (SOC) (ARR = 0.73, 0.47−0.99, *p*<0.05) [42]. Another study reported a marked improvement in condom use assessed at the 3‐month follow‐up among female adults, with significantly higher usage in the lottery incentive group (75%) compared to the SOC group (40%) (OR = 4.5, 1.43−14.1, *p*<0.05); however, this effect persisted for 6 months and then diminished [43].

**TABLE 1:** Behavioural economics‐informed interventions for STBBIs (*N* = 6).

Study	Participants	Age (years)	Gender	Comparisons	Results (outcomes)	Test statistics
De Walque et al. [42]	Young adults	Mean (SD): 27.4 (5.6)	Male: 49.8% Female: 50.2%	T1: Incentive (monetary) $10 T2: Incentive (monetary) $20 C: Standard of care	**Primary**: **Combined prevalence of four STIs at 12 months** ^a^ T1: 12.8% (79/617) T2: 9.7% (57/588) C: 12.1% (126/1041)	Combined prevalence of four STIs at 12 months ^a^ T1 versus C: ARR (95% CI) = 1.06 (0.75−1.38) T2 versus C: ARR (95% CI) = 0.73 (0.47−0.99)*
Galárraga et al. [43]	Female adults	Mean (SD): 26.9 (5.9)	Female: 100%	T: Incentives (lottery) C: Standard of care	**Secondary**: **Condom use at 3 months** T: 75% (30/40) C: 40% (8/20) **Condom use at 6 months** T: 72% (20/28) C: 50% (5/10)	Condom use at 3 months T versus C: OR (95% CI) = 4.50 (1.43−14.1)* Condom use at 6 months T versus C: OR (95% CI) = 2.50 (0.57−11.1)
Thirumurthy et al. [44]	Uncircumcised male adults	Mean (SD): 34.4 (6.7)	Male: 100%	T1: Incentives ($2.50 food voucher) T2: Incentives ($8.75 food voucher) T3: Incentives ($15 food voucher) C: Standard of care	**Primary**: **Voluntary MMC uptake within 2 months** T1 = 1.9% (7/374) T2 = 6.6% (25/381) T3 = 9.0% (34 of 377) C: 1.6% (6/370)	Voluntary MMC uptake within 2 months T1 versus C: AOR (95% CI) = 1.1 (0.4−3.3) T2 versus C: AOR (95% CI) = 4.3 (1.7−10.7)** T3 versus C: AOR (95% CI) = 6.2 (2.6−15.0)***
Thirumurthy et al. [45]	Uncircumcised male adults	Mean (SD): 29 (5.9)	Male: 100%	T1: Incentives (lottery) T2: Fixed compensation C: Standard of care	**Primary**: **Voluntary MMC uptake within 3 months** T1 = 3.3% (10/302) T2 = 8.4% (26/308) C = 1.3% (4/299)	Voluntary MMC uptake within 3 months T1 versus C: AOR (95% CI) = 2.5 (0.8−8.1) T2 versus C: AOR (95% CI) = 7.1 (2.4−20.8)** T1 versus T2: P for Equality Test: AOR**
Stevens et al. 2024	Pregnant adolescent girls	Mean (SD): 18 (1.2)	Female: 100%	T: Providing reminders C: Standard of care	**Secondary**: **Number of HPV shots at 12 months** T: Mean (SD) = 2.21 (1.20) C: Mean (SD) = 2.14 (1.14) **Number of HPV shots at 18 months** T: Mean (SD) = 2.31 (1.13) C: Mean (SD) = 1.86 (1.30)	Number of HPV shots at 12 months T versus C: Differences = 0.02 Number of HPV shots at 18 months T versus C: Differences = 0.41+
Tang et al. [46]^b^	MSM	16−20: 29% 21–25: 36%	Male: 100%	T: Crowdsourced educational materials C: Social marketing interventions	Primary: Self‐reported condomless sex in the 3 weeks T: 33.6% (146/434) C: 32.3% (153/473) Self‐reported condomless sex in the 3 months T: 52.1% (196/376) C: 49.6% (206/415) Secondary: HIV testing in the 3 weeks T: 17.3% (75/434) C: 18.0% (85/473) Syphilis testing in the 3 weeks T: 13.4% (58/434) C: 11.0% (52/473) HIV testing in the 3 months T: 38.0% (143/376) C: 34.5% (143/415) Syphilis testing in the 3 months T: 22.6% (85/376) C: 22.2% (92/415)	Self‐reported condomless sex in the 3 weeks T versus C: Difference (95% CI): 1.3% (CI: −4.8% to 7.4%) Self‐reported condomless sex in the 3 months T versus C: Difference (95% CI): 2.5% (95% CI: −4.5% to 9.5%) HIV testing in the 3 weeks T versus C: Difference (95% CI) = −0.7 (−5.6 to 4.3) Syphilis testing in the 3 weeks T versus C: Difference (95% CI) = 2.4% (−1.9% to 6.6%) HIV testing in the 3 months T versus C: Difference (95% CI): 3.5% (−3.1% to 10.3%) Syphilis testing in the 3 months T versus C: Difference (95% CI): 0.4% (−5.4% to 6.3%)

Abbreviations: AOR, adjusted odds ratio; ARR, adjusted relative risk; HIV, human immunodeficiency virus; HPV, human papillomavirus; MMC, medical male circumcision; MSM, men who have sex with men; OR, odds ratio; SD, standard deviation; STBBIs, sexually transmitted and blood‐borne infections; STI, sexually transmitted infection.

^a^ Monetary incentives were used to promote safer sexual practices, reducing the prevalence of four STIs: Chlamydia trachomatis, Neisseria gonorrhoeae, Trichomonas vaginalis and Mycoplasma genitalium.

^b^ Non‐inferiority design.

*p*<0.1+; *p*≤0.05*, *p*≤0.01**, *p*≤0.001***.

Voucher‐based incentives were also effective in increasing MMC uptake. Studies found significantly higher uptake in intervention groups receiving USD 8.75 vouchers (AOR = 4.3, 1.7−10.7, *p*<0.01) and USD 15 vouchers (AOR = 6.2, 2.6−15.0, *p*<0.001) compared to SOC in Kenya [44]. However, fixed monetary compensation was more effective than lottery incentives in improving MMC uptake (*p*<0.01) [45]. A non‐inferiority trial evaluating the impact of crowdsourced educational materials compared to social marketing interventions found no statistically significant differences between these approaches for either primary outcomes related to sexual behaviours or secondary outcomes assessing HIV/STI testing uptake [46]. Taken together, these findings suggest that STBBI prevention‐related behaviours may be more responsive to incentive‐based approaches when the target action is one‐off or intermittent, although effects may not persist over time.

### 3.4 Impact of Behavioural Economics‐Informed Interventions on STBBI Care Engagement

Behavioural economics‐informed interventions demonstrated varied effectiveness in improving engagement across the STBBI care cascade, with marked successes in testing uptake (Table 2). For HIV testing, opt‐out and active‐choice default strategies substantially increased uptake, significantly outperforming opt‐in approaches (*p*<0.001) [47] and yielding uptake rates of 55.3% (active‐choice, *p*<0.01) and 67.7% (opt‐out, *p*<0.01) compared with SOC [27]. HIV testing uptake was also effectively increased by monetary incentives (USD 5: 62.1%, *p*<0.01; USD 10: 66.6%, *p*<0.01 vs. SOC) [27], immediate monetary incentives (β = 43.63, 33.27−54.00, *p*<0.05 vs. information alone) [28], voucher incentives (*p*<0.05 vs. SOC) [29] and gain‐framed messaging (AOR = 1.89, 1.21−2.95, *p*<0.01) [31]. For HIV retesting, monetary incentives consistently improved uptake compared with SOC (OR 4.8, 3.0−7.7, *p*<0.001) and deposit contracts (OR = 5.5, 3.3−9.1, *p*<0.001) [33].

**TABLE 2:** Behavioural economics‐informed interventions in STBBI care engagement (*N* = 23).

Study	Participants	Age (years)	Gender	Comparisons	Results (outcomes)	Test statistics
Hunter et al. [29]	Adolescent girls and young women (AGYW)	15−24: 100%	Female: 100%	T: Incentives (voucher) C: Standard of care	**Primary**: **HIV self‐test kits uptake** T: Mean per shop (SD) = 145.6 (107.6) C: Mean per shop (SD) = 59.6 (30.4) **Condom use** T: Mean per shop (SD) = 128.9(142.7) C: Mean per shop (SD) = 12.3(25.8) **Linkage to HIV care** T: Mean per shop (SD) = 8.2 (20.0) C: Mean per shop (SD) = 0.2 (0.4)	HIV self‐test kits uptake T versus C: 145.6 (107.6) versus 59.6 (30.4)* Condom use: T versus C: 128.9(142.7) versus 12.3 (25.8)* Linkage to HIV care T versus C: 8.2 (20.0) versus 0.2 (0.4)
Montoy et al. [27]	Patients of the medical facility	Median (IQR): 40 (30–52)	Male: 59.6% Female: 40.4%	T1: Incentives (monetary); T2: Defaults (active‐choice, opt‐in, opt‐out) C: Standard of care	**Primary**: **HIV testing uptake** T1‐$1: 52.6% T1‐$5: 62.1% T1‐$10: 66.6% C1: No incentive: 51.6% T2: Active‐choice: 55.3% T2: Opt‐out: 67.7% C2: Opt‐in: 43.8%	HIV testing uptake T1‐$1 versus C1: Difference (SE) = 0.011 (0.016) T1‐$5 versus C1: Difference (SE) = 0.105 (0.017)** T1‐$10 versus C1: Difference (SE) = 0.15 (0.016)** T2: Active‐choice versus C2: Difference (SE) = 0.115 (0.013)** T2: Opt‐out versus C2: Difference (SE) = 0.239 (0.013)**
Montoy et al. [47]	Patients of the medical facility	Median (IQR): 42 (31−53)	Male: 60.1% Female: 39.9%	T1: Defaults (active choice) T2: Defaults (opt‐out) C: Opt‐in	**Primary**: **HIV testing uptake** T1: 51.3% (835/1628) T2: 65.9% (1031/1565) C: 38.0% (611/1607)	HIV testing uptake T1 versus C: Difference (95% CI) = 13.3% (9.8−16.7)*** T2 versus C: Difference (95% CI) = 27.9% (24.4−31.3)***
Smith et al. [31]	Male adults	Median (IQR): 35 (27–45)	Male: 100%	T: Framing (gain‐framed) C: Standard of care	**Primary**: **Invited and tested for HIV** T: 22% (112/504) C: 14% (76/544) **Secondary**: **Invited and came for HIV testing** T: 25% (125/504) C: 13% (68/544) **Linked to ART** T: 43% (3/7) C: 67% (2/3)	Invited and tested for HIV T versus C: AOR (95% CI) = 1.89 (1.21−2.95)** Invited and came for HIV testing T versus C: AOR (95% CI) = 2.03 (1.48−2.78)+ Linked to ART: T versus C: AOR (95% CI) =—(Mid‐P exact = 0.29)
Chamie et al. 2018	Male adults	Mean (SD): 36.88 (15.71)	Male: 100%	T1: Incentives (lottery) T2: Framing (loss‐framed) C: Framing (gain‐framed)	**Primary**: **Tested for HIV** T1: 78% (648/833) T2: 77% (638/833) C: 74% (638/861)	Tested for HIV T1 versus C: 78% versus74%+ T2 versus C: 77% versus 74%
Chamie et al. [33]	Adults	Median (IQR): 25 (22−29)	Male: 55.92% Female: 44.08%	T1: Incentives (monetary) T2: Commitment (deposit contracts) C: Standard of care	**Primary**: **HIV retest uptake at 3 and 6 months** T1: 52% (89/172) T2: 16% (28/172) C: 18% (33/180)	HIV retest uptake at 3 and 6 months T1 versus C: OR (95% CI) = 4.8 (3.0−7.7)*** T2 versus C: OR (95% CI) = 0.87 (0.5−1.5) T1 versus T2: OR (95% CI) = 5.5 (3.3−9.1)***
Reina Ortiz et al. [61]	Pregnant women	18–27: 67.8% 28–47: 32.2%	Female: 100%	T1: Soft‐commitment T2: Incentives (monetary) C: Information only	**Primary**: **HIV testing uptake** T1: 1.4% (2/138) T2: 66.7% (118/177) C: 9.1% (10/110)	HIV testing uptake T1 versus C: AOR (95% CI) = 0.14 (0.03−0.68)** T2 versus C: AOR (95% CI) = 17.06 (8.14−35.77)***
Macis et al. [28]	Adults	18−22: 27.0% 23–32: 36.0% 33–47: 25.8% ≥48: 11.3%	Male: 41.3% Female: 58.7%	T1: Soft‐commitment T2: Immediate‐incentive (monetary) T3: Delayed‐incentive (monetary) C:Information alone	**Primary**: **HIV testing uptake** T1: 11% (333/3024) T2: 66.3% (534/805) T3: 8.7% (95/1090) C: 12.21% (343/2801)	HIV testing uptake T1 versus C: β (95% CI) = −1.88 (−3.86 to 1.09) T2 versus C: β (95% CI) = 43.63 (33.27−54.00)* T3 versus C: β (95% CI) = 1.88 (−2.41 to 6.16)
Brown et al. [35]	Online HIV self‐testers	16–24: 36.08% 25–34: 35.87% 35–49: 20.6% 50–64: 6.62% ≥ 65: 0.82%	MSM: 65.55% WSW: 3.28% Trans: 0.63% Heterosexuals: 30.54%	T1: Planning prompts T2: BE reminders T3: Planning prompts + BE reminders C: Standard reminders	**Primary**: **HIV testing kits returned (%)** T1: 52.92% (1204/2275) T2: 55.19% (1222/2214) T3: 56.29% (1276/2267) C: 52.39% (1175/2243)	HIV testing kits returned (%) T1 versus C: OR (95% CI) = 1.02 (0.91−1.15) T2 versus C: OR (95% CI) = 1.12 (1.00−1.32)+ T3 versus C: OR (95% CI) = 1.17 (1.04−1.32)** T2 versus T1: OR (95% CI) = 1.10 (0.97−1.23) T3 versus T1: OR (95% CI) = 1.15 (1.02−1.29)* T3 versus T2: OR (95% CI) = 1.05 (0.93−1.18)
Kavanagh et al. [36]	Male adults	Mean (SD): 38.6 (15.9)	Male: 100%	I: Planning prompts C: Received a calendar	**Primary**: **HIV testing uptake** T: 77.1% (928/1203) C: 74.9% (868/1159)	HIV testing uptake T versus C: AD% (95 CI%) = 2.5% (–0.9 to 5.9)
Tang et al. [40]^a^	MSM and transgender people	16−20: 32.5% 21–25: 36.9% 26–30: 15.5% 31–35: 8.9% ≥36: 6.2%	Male: 95% Transgender: 5%	T: Crowdsourced educational materials C: Health marketing	**Primary**: **Self‐reported first‐time HIV testing** T: 37.1% (114/307) C: 35% (111/317) **Secondary**: **Cost per first‐time HIV test** T: US $131 C: US $238 **Cost per new HIV diagnosis** T: US $415 C: US $799	Self‐reported first‐time HIV testing T versus C: RD (95% CI) = 2.1% (−5.4% to 9.7%) Secondary: Cost per first‐time HIV test T versus C: Ratio = 0.55 Cost per new HIV diagnosis T versus C: Ratio = 0.52
Lin et al. [49]	MSM	<30: 64.3% ≥30: 35.7%	Male: 100%	T: Crowdsourced educational materials C: Standard of care	**Primary**: **HIV testing uptake at 3 months** T: 49.8% (139/279) C: 43.8% (183/418) **HIV testing uptake at 6 months** T: 55.6% (148/266) C: 43.6% (178/408) **HIV testing uptake at 9 months** T: 71.9% (189/263) C: 51.1% (206/403) **HIV testing uptake at 12 months** T: 64.3% (171/266) C: 48.4% (182/397)	HIV testing uptake at 3 months T versus C: 49.8% versus 43.8% HIV testing uptake at 6 months T versus C: 55.6% versus 43.6% ** HIV testing uptake at 9 months T versus C: 71.9% versus 51.1% *** HIV testing uptake at 12 months T versus C: 64.3% versus 48.4% *** Testing for HIV in the previous 3 months T versus C: RR (95% CI) = 1.69 (0.87−3.27)
Yan et al. [50]	PLWH	Mean (SD): 30.2 (9.24)	Male: 100%	T: Crowdsourced educational materials C: Standard of care	**Primary**: **Proportions of partners getting HIV tested** T: 38% (65/171) C: 27% (24/89) **Secondary**: **Proportions of partners testing HIV positive out of tested partners** T: 23% (15/65) C: 13% (3/24)	Proportions of partners getting tested out of reported partners T versus C: RD (95% CI) = 11% (−2% to 24%) Proportions of partners testing positive out of tested partners T versus C: RD (95% CI) = 10% (−9% to 30%)
Mehta et al. [30]	Patients of the medical facility	Mean (SD): 62.8 (6.0)	Male: 45.2% Female: 54.8%	T1: Defaults + Letter T2: Norms C1: Letter only C2: Standard of care	**Primary**: **HCV screening at 4 months** T1: 43.1% (357/829) T2: 13.6% (1341/9833) C1: 19.2% (156/813) C2: 14.6% (1431/9828) **Secondary**: **HCV screening at 12 months** T1: 56.2% (466/829) T2: 28.1% (2765/9833) C1: 34.3% (279/813) C2: 29.0% (2855/9828)	HCV screening at 4 months T1 versus C1:Difference (95% CI) = 23.9% (19.6−28.2)*** T2 versus C2:Difference (95% CI) = −1.0% (−1.9 to 0.0)+ HCV screening at 12 months T1 versus C1:Difference (95% CI) = 21.9% (17.2−26.6)*** T2 versus C2:Difference (95% CI) = −0.9% (−2.2 to 0.3)
Niza et al., [32]	College students	18−24: 100%	Not reported	T1: Incentives (voucher) − Framing (gain‐framed) T2: Incentives (voucher) − Framing (loss‐framed) T3: Incentives (lottery) − Framing (gain‐framed) T4: Incentives (lottery) − Framing (loss‐framed) C: No incentives	**Primary**: **Chlamydia screening kits returned (%)** T1: 23.7% T2: 21.6% T3: 3.6% T4: 2% C: 1.5%	Chlamydia screening kits returned (%) T1+T2+ T3+T4 versus C: β (95% CI) = 2.719 (1.159−4.279)*** T3+T4 versus T1+T2: β (95% CI) = −1.180 (−1.822 to −0.539)*** T1+T3 versus T2+T4: β (95% CI) = 0.117 (−0.201 to 0.436)
Fitzpatrick et al. [51]	MSM	Mean (SD): 25.6 (7)	Male: 100%	T: Crowdsourced educational materials C: Standard of care	**Primary**: **Confirmed HBV and HCV test uptake at 4 weeks** T: 7.9% (22/280) C: 8.0% (22/276) **Secondary**: **Self‐reported HBV and HCV test uptake at 4 weeks** T: 16.1% (45/280) C: 18.8% (52/276) **HBV vaccination uptake at 4 weeks** T: 6.4% (18/280) C: 7.6% (21/276) **HIV test uptake at 4 weeks** T: 40.7% (114/280) C: 37.3% (103/276) **Chlamydia test uptake at 4 weeks** T: 5.0% (14/280) C: 4.7% (13/276) **Gonorrhoea test uptake at 4 weeks** T: 6.1% (17/280) C: 6.5% (18/276) **Syphilis test uptake at 4 weeks** T: 22.1% (62/280) C: 19.6% (54/276) **Visit with a physician after hepatitis test** T: 9.6% (27/280) C: 9.4% (26/276)	Confirmed HBV and HCV test uptake at 4 weeks T versus C: OR (95% CI) = 0.98 (0.53−1.82) Self‐reported HBV and HCV test uptake at 4 weeks T versus C: OR (95% CI) = 0.82 (0.53−1.28) HBV vaccination uptake at 4 weeks T versus C: OR (95% CI) = 0.83 (0.43−1.60) HIV test uptake at 4 weeks T versus C: OR (95% CI) = 1.15 (0.82−1.62) Chlamydia test uptake at 4 weeks T versus C: OR (95% CI) = 1.06 (0.49−2.31) Gonorrhoea test uptake at 4 weeks T versus C: OR (95% CI) = 0.93 (0.47−1.84) Syphilis test uptake at 4 weeks T versus C: OR (95% CI) = 1.17 (0.78−1.76) Visit with a physician after hepatitis test T versus C: OR (95% CI) = 1.03 (0.58−1.81)
Wong et al. [52]	Primary care patients	Mean (SD): 42.7 (11.1)	Male: 42.3%, Female: 57.5% Other: 0.3%	T: Crowdsourced educational materials C: Standard of care	**Primary**: **Confirmed HBV testing at 4 weeks** T: 58.1% (256/376) C: 69.0% (258/374) **Confirmed HCV testing at 4 weeks** T: 57.7% (217/376) C: 56.7% (212/374) **Confirmed both HBV and HCV tests at 4 weeks** T: 49.5% (186/376) C: 50.5% (189/374) **Secondary**: **Confirmed HBV positives at 4 weeks** T: 3.7% (14/376) C: 2.1% (8/374) **Confirmed HCV positive at 4 weeks** T: 0.3% (1/376) C: 0.5% (2/374) **Sought medical care after receiving HBV results (self‐report) at 4 weeks** T: 29.0% (108/376) C: 33.4% (126/374) **Sought medical care after receiving HCV results (self‐report) at 4 weeks** T: 22.5% (84/376) C: 27.1% (102/374) **Self‐reported HBV vaccination at 4 weeks** T: 5.9% (22/376) C: 4.8% (18/374)	Primary: Confirmed HBV testing at 4 weeks T versus C: OR (95% CI) = 1.23 (0.81−1.89) Confirmed HCV testing at 4 weeks T versus C: OR (95% CI) = 1.24 (0.87−1.76) Confirmed both HBV and HCV tests at 4 weeks T versus C: OR (95% CI) = 1.20 (0.85−1.71) Confirmed HBV positives at 4 weeks T versus C: OR (95% CI) = 1.77 (0.75−4.48) Confirmed HCV positive at 4 weeks T versus C: OR (95% CI) = 0.50 (0.02−5.20) Sought medical care after receiving HBV results (self‐report) at 4 weeks T versus C: OR (95% CI) = 0.87 (0.62−1.22) Sought medical care after receiving HCV results (self‐report) at 4 weeks T versus C: OR (95% CI) = 0.87 (0.61−1.24) Self‐reported HBV vaccination at 4 weeks T versus C: OR (95% CI) = 1.32 (0.69−2.54)
Yang et al. [38]	MSM	Mean (SD): 28.1 (7.1)	Male: 100%	T1: Pay‐it‐forward T2: Pay‐what‐you‐want C: Standard of care	**Primary**: **Dual chlamydia and gonorrhoea testing uptake at the intervention visit** T1: 56% (57/101) T2: 46% (46/100) C: 18% (18/100) **Secondary**: **The economic cost per person tested** T1: US $19.72 T2: US $21.02 C: US $34	Dual chlamydia and gonorrhoea testing uptake at the intervention visit T1 versus C: AD (95% CI) = 39.0% (28.3%−100%) *** T2 versus C: AD (95% CI) = 27.8% (15.3%−100%) *** The economic cost per person tested T1 versus C: ICER = US$12.96 T2 versus C: ICER = US$12.68 T1 versus T2: ICER = US$14.27
Tang et al. [48]	Female sex workers	Mean (SD): 34.7 (9.0)	Female: 100%	T: Pay‐it‐forward C: Standard of care	**Primary**: **Dual chlamydia and gonorrhoea testing uptake at the intervention visit** T: 82.1% (197/240) C: 4.2% (10/240) **Secondary**: **The economic cost per person tested** T: US $41.80 C: US $42.24	Dual chlamydia and gonorrhoea testing uptake at the intervention visit T versus C: AD (95% CI) = 76.7% (70.8%−100)*** The economic cost per person tested T versus C: ICER = US $ −2.79
Zhang et al. [39]	MSM	Median (IQR): 29 (25–37)	Male: 100%	T: Pay‐it‐forward C: Standard of care	Primary: **Dual HBV and HCV testing uptake at the intervention visit** T: 59.4% (95/160) C: 25.3% (41/162) **Secondary**: **The economic cost per person tested** T: US $3.99 C: US $3.78	The proportion of dual HBV and HCV testing uptake T versus C: AD (95% CI) = 35.2% (24.1%−46.3%)*** The economic cost per person tested T versus C: ICER (not reported) Dominated.
Bokolo et al. [10]	Adults	18−24: 16.5% 25–34: 50.3% 35–44: 26.7% ≥ 45: 5.8% Missing: 0.6%	Male:59.1% Female: 40.1% Trans/other: 0.2% Missing: 0.6%	T1: Framing (gain‐framed) T2: Incentive (care voucher) C: Standard of care	**Primary**: **HIV clinic visits within 4 months** T1: 4.0% (153/3804) T2: 4.1% (158/3829) C: 3.6% (137/3802)	HIV clinic visits within 4 months T1 versus C: AOR (95% CI) = 1.02 (0.79−1.33) T2 versus C: AOR (95% CI) = 1.08 (0.84−1.40)
Brown et al. [35]	PLWH	Mean: 33.0	Male: 36.0% Female: 64.0%	T: Incentive (voucher) C: Standard of care	**Primary**: **Linkage to HIV care within 3 months** T: 63% (29/46) C: 72.5% (29/40) **ART initiation within 3 months** T: 39% (18/46) C: 45% (18/40)	Linkage to HIV care within 3 months T versus C: AOR (95% CI) = 0.70 (0.26−1.91) ART initiation within 3 months T versus C: AOR (95% CI) = 0.67 (0.26−1.78)
Barnabas et al. [53]	PLWH	18−24: 11% 25–29: 12% 30–49: 69% ≥50: 8%	Male:100%	T: Incentives (lottery) C: Standard of care	Primary: Linkage to the ART clinic T: 93% (52/56) C: 77% (58/75) Initiated ART T: 93% (52/56) C: 76% (57/75) HIV viral suppression (viral load <20 copies/mL) T: 66% (37/56) C: 59% (44/75)	Linkage to the ART clinic T versus C: ARR (95% CI) = 1.21 (0.83−1.76) Initiated ART T versus C: ARR (95% CI) = 1.23 (0.84−1.79) HIV viral suppression (viral load <20 copies/mL) T versus C: ARR (95% CI) = 1.13 (0.73−1.75)

Abbreviations: AD, adjusted difference; AGYW, adolescent girls and young women; AOR, adjusted odds ratio; ART, antiretroviral therapy; BE, behavioural economics; HBV, hepatitis B virus; HCV, hepatitis C virus; HIV, human immunodeficiency virus; ICER, incremental cost‐effectiveness ratio; IQR, interquartile range; MSM, men who have sex with men; OR, odds ratio; PLWH, people living with HIV; RD, risk difference; SD, standard deviation; STBBIs, sexually transmitted and blood‐borne infections; WSW, women who have sex with women.

*p*<0.1+; *p*≤0.05*, *p*≤0.01**, *p*≤0.001***.^a^

^a^ Non‐inferiority design.

Similar positive effects were observed for other STBBIs. For HCV screening, integrating default options with reminders significantly increased rates at 4 months (43.1% vs. 19.2% SOC, *p*<0.001) and 12 months after intervention (56.2% vs. 34.3% SOC, *p*<0.001) [30]. For chlamydia screening, combining incentives with framing significantly increased kit returns (β = 2.719, 1.159−4.279, *p*<0.001), although voucher‐based framing was more effective than lottery‐based approaches (β = –1.18, –1.822 to –0.539, *p*<0.001) [32]. Among MSM, pay‐it‐forward and pay‐what‐you‐want significantly increased dual chlamydia and gonorrhoea testing (AD = 39.0%, 28.3%−100%, *p*<0.001; and AD = 27.8%, 15.3%–100%, *p*<0.001, respectively, vs. SOC) [38]. A pay‐it‐forward approach also markedly boosted dual chlamydia and gonorrhoea testing among female sex workers (AD = 76.7%, 70.8%–100%, *p*<0.001; cost‐saving vs. SOC) [48] and enhanced dual HBV and HCV testing among MSM (AD = 35.2%, 24.1%−46.3%, *p*<0.001; cost‐effective vs. SOC) [39].

The effectiveness of crowdsourced educational materials was inconsistent. For HIV testing among MSM and transgender people, crowdsourced materials were non‐inferior to health marketing and more cost‐effective [40]. Another study in MSM reported significantly higher HIV testing with crowdsourced materials at 6, 9 and 12 months (*p*<0.01, *p*<0.001, *p*<0.001, respectively, vs. SOC) [49]. However, among people living with HIV (PLWH), these materials did not significantly increase partners’ HIV testing uptake [50], nor did interventions relying solely on crowdsourced materials show significant improvements for other STBBI testing in recent trials [51, 52].

Behavioural economics‐informed interventions showed less promising results in improving linkage to HIV care. Lottery incentives did not significantly improve linkage to antiretroviral therapy (ART) clinics or ART initiation among PLWH [53]. Similarly, voucher incentives failed to significantly improve linkage to HIV care or ART initiation within 3 months [54]. For linkage to hepatitis care, crowdsourced educational materials also did not significantly increase visits with a physician after hepatitis testing (OR = 1.03, 0.58−1.81) [51] or seeking medical care after receiving HBV (OR = 0.87, 0.62−1.22) or HCV results (OR = 0.87, 0.61−1.24) [52]. Taken together, these findings suggest that behavioural economics‐informed interventions were generally more effective in improving STBBI testing uptake than linkage to care or treatment initiation, with more consistent benefits observed for screening‐related outcomes across infections and populations.

### 3.5 Impact of Behavioural Economics‐Informed Interventions on STBBI Treatment Adherence

Behavioural economics‐informed interventions demonstrated significant impacts on HIV treatment adherence, with most studies (10/11) employing incentive‐based strategies to improve retention in care, adherence to ART and viral suppression among PLWH (Table 3). Retention in care was significantly improved by monetary incentives, particularly among pregnant women living with HIV. At 6 weeks postpartum, monetary incentives increased retention rates (ARR = 1.13, 1.02−1.26, *p*<0.05), with even greater improvement observed among women who attended all clinic visits and received comprehensive services (ARR = 1.31, 1.12−1.54, *p*<0.001) [55].

**TABLE 3:** Behavioural economics‐informed interventions in STBBI treatment adherence (*N* = 11).

Study	Participants	Age	Gender	Comparisons	Results (outcomes)	Test statistics
Yotebieng et al. [55]	PLWH (pregnant women)	Median (IQR): 29.0 (25.0–34.0)	Female: 100%	T: Incentives (monetary) C: Standard of care	**Primary**: **Retention in HIV care at 6 weeks postpartum** T: 80.6% (174/216) C: 72.4% (157/217) **Attended each clinic visit and received services** T: 67.6% (146/216) C: 53.5% (116/217)	Retention in HIV care at 6 weeks postpartum T versus C: ARR (95% CI) = 1.13 (1.02−1.26)* Attended each clinic visit and received services T versus C: ARR (95% CI) = 1.31 (1.12−1.54)***
Fahey et al. [60]	PLWH	Median (IQR): 34.7 (28.4−42.2)	Male: 37.7% Female: 62.3%	T1: Incentives (monetary) $4.50 T2: Incentives (monetary) $10 C: Standard of care	**Primary**: **Retained in HIV care and viral suppression (<1000 copies per mL) at 6 months** T1: 82.9% (143/172) T2: 86.1% (150/174) C: 73.0% (134/184) **Secondary**: **Retention on ART at 6 months** T1: 88.4% (152/172) T2: 90.8% (158/174) C: 83.7% (154/184) **HIV viral suppression (<1000 copies per mL) at 6 months** T1: 93.8% (143/152) T2: 94.9% (150/158) C: 87.1% (134/154)	Retained in HIV care and viral suppression (<1000 copies per mL) at 6 months T1 versus C: RD (95% CI) = 9.8 (1.2−18.5)* T2 versus C: RD (95% CI) = 13.0 (4.5−21.5)** Retention on ART at 6 months T1 versus C: RD (95% CI) = 4.7 (−2.5 to 11.8) T2 versus C: RD (95% CI) = 7.1 (0.3−13.9)* HIV viral suppression (<1000 copies per mL) at 6 months T1 versus C: RD (95% CI) = 6.7 (−0.2 to 13.6)+ T2 versus C: RD (95% CI) = 7.8 (0.9−14.7)*
Silverman et al. [56]	PLWH	Mean (SD): 47 (9.5)	Male: 54% Female: 46%	T: Incentives (monetary) C: Standard of care	**Primary**: **HIV viral suppression** T: 76.9% (40/52) C: 41.5% (21/50) **Secondary**: **Self‐reported ART adherence (> 90%)** T: 69.9% (36/52) C: 45.2% (23/50)	HIV viral suppression T versus C: OR (95% CI) = 14.3 (4.3−47.7)** Self‐reported ART adherence (> 90%) T versus C: OR (95% CI) = 5.9 (1.9−18.2)**
Alsan et al. [34]	PLWH	Median: 43.97	Not reported	T1: Incentives (monetary)—provider visit incentive T2: Incentive choice—monetary or commitment contract C: Standard of care	**Primary**: **HIV viral suppression at fifth visit** T1: 42% (8/19) T2: 38% (8/21) C: 34% (24/70) **Secondary**: **HIV viral suppression at sixth visit** T1: 68% (13/19) T2: 43% (9/21) C: 41% (29/70)	HIV viral suppression at fifth visit T2 versus T1: AOR (95% CI) = 1.57 (0.25−9.92) T2 versus C: AOR (95% CI) = 1.44 (0.46−4.49) HIV viral suppression at sixth visit T2 versus T1: AOR (95% CI) = 1.57 (0.25−9.92) T2 versus C: AOR (95% CI) = 3.93 (1.19−13.04)*
Thirumurthy et al. 2019	PLWH	Median (IQR): 37 (31–45)	Male: 44% Female: 56%	T1: Incentives (monetary) C: Standard of care	**Primary**: **HIV viral suppression at 6 months** T: 84% (168/201) C: 82% (156/191)	HIV viral suppression at 6 months T versus C: AOR (95% CI) = 1.14 (0.66−1.97)
Linnemayr et al. [37]	PLWH	Mean (SD): 33.9 (9.66)	Male: 34.2% Female: 65.8%	T1: Messages (salience) T2: Incentives (monetary) C: Standard of care	**Primary**: **Mean adherence to ART at 1–3 months (intervention period)** T1: 75.1% (37/49) T2: 81.0% (46/57) C: 68.5% (34/49) **Mean adherence to ART at 4–6 months** T1: 61.3% (30/49) T2: 71.7% (41/57) C: 57.5% (28/49) **Mean adherence to ART at 7–9 months** T1: 53.0% (26/49) T2: 66.4% (38/57) C: 51.5% (25/49)	Mean adherence to ART at 1–3 months (intervention period) T1 versus C: Adjusted β (95% CI) = 7 (−2.8 to 16.7) T2 versus C: Adjusted β (95% CI) = 12.2 (2.2−22.2)* Mean adherence to ART at 4–6 months T1 versus C: Adjusted β (95% CI) = 4.9 (−8.4 to 18.1) T2 versus C: Adjusted β (95% CI) = 14.2 (1.1−27.2)* Mean adherence to ART at 7–9 months T1 versus C: Adjusted β (95% CI) = 1.4 (−14.1 to 16.9) T2 versus C: Adjusted β (95% CI) = 14.1 (−0.2 to 28.5)+
Linnemayr et al. [57]	PLWH	Mean (SD): 37.83 (1.43)	Male: 37% Female: 63%	T1: Clinic‐linked incentives (lottery) T2: Adherence‐linked incentives (lottery) C: Standard of care	**Primary**: **Mean adherence to ART** T1+T2: 78.6 (173/329) T1: 80.1% (87/109) T2: 77.1% (86/111) C: 76.1% (83/109) **HIV viral suppression** T1+T2: 82% (180/329) T1: 81.8% (89/109) T2: 82.1% (91/111) C: 85.6% (93/109)	Mean adherence to ART T1+T2 versus C: AD (95% CI) = 0.032 (−0.008 to 0.072) T1 versus C: AD (95% CI) = 0.024 (−0.020 to 0.069) T2 versus C: AD (95% CI) = 0.039 (‐0.007−0.086)+ HIV viral suppression T1+T2 versus C: AD (95% CI) = −0.021 (−0.112 to 0.069) T1 versus C: AD (95% CI) = −0.036 (−0.142 to 0.07) T2 versus C: AD (95% CI) = −0.006 (−0.111 to 0.098)
Linnemayr et al. [58]	PLWH	Mean (SD): 39 (10.3)	Male: 37% Female: 63%	T1: Clinic‐linked incentives (lottery) T2: 90% adherence‐linked incentive (lottery) C:Standard of care	**Primary**: **Mean ART adherence over 9 months** T1+T2: 87.5% (86/98) T1: 88.3% (41/46) T2: 86.7% (45/52) C: 80.9% (39/48) **Mean ART adherence (> 90%) over 9 months** T1+T2: 63.3% (62/98) T1: 60.9% (28/46) T2: 65.4% (34/52) C: 39.6% (19/48)	Mean ART adherence over 9 months T1+T2: versus C: Adjusted β (95% CI) = 5.2 (−1.6 to 11.9) T1 versus C: Adjusted β (95% CI) = 6.4 (−0.7 to 13.5)+ T2 versus C: Adjusted β (95% CI) = 4.7 (−2.9 to 12.3) Mean ART adherence (> 90%) over 9 months T1+T2: versus C: Adjusted β (95% CI) = 21.2 (3.0−39.4)* T1 versus C: Adjusted β (95% CI) = 20.5 (−1.3 to 42.3)+ T2 versus C: Adjusted β (95% CI) = 24.8 (4.0−45.7)*
Stecher et al. [59]	PLWH	Mean: 39	Male: 36% Female: 64%	T1: Clinic‐linked incentives (lottery) T2: Adherence‐linked incentives (lottery) C: Standard of care	**Primary**: **Mean ART adherence over 20 months** T1: 0.83 T2: 0.84 C: 0.81	Mean ART adherence over 20 months T1 versus C:Adjusted β (SE) = 0.0280 (0.033) T2 versus C:Adjusted β (SE) = 0.0539 (0.031)
Bien‐Gund et al. 2021	PLWH	Mean (SD): 44.2 (10.5)	Male: 55.2% Female: 41.4% Trans: 3.4%	T: Incentives (lottery) C: Standard of care	**Primary**: **HIV viral suppression (viral load<400 copies/mL)** T: 4/15 (27%) C: 4/14 (29%)	HIV viral suppression (viral load<400 copies/mL) T versus C: Difference (95% CI) = 1.9% (−34.5 to 30.7)
MacCarthy et al. [63]	PLWH (youth)	15−24: 100%	Male: 20.4% Female: 79.6%	T1: Received own adherence‐level texts T2: Norms (received own and peers’ adherence‐level texts) C: Standard of care	Primary: Mean ART adherence over 9 months T1: 76.5% T2: 82.5% C: 81.1%	Mean ART adherence over 9 months T1 versus C: AD (95% CI) = −3.8 (−9.9 to 2.3) T2 versus C: AD (95% CI) = 2.4 (−3.0 to 7.9)

Abbreviations: AD, adjusted difference; AOR, adjusted odds ratio; ARR, adjusted relative risk; ART, antiretroviral therapy; CI, confidence interval; HIV, human immunodeficiency virus; IQR, interquartile range; OR, odds ratio; PLWH, people living with HIV; RD, risk difference; SD, standard deviation; SE, standard error; STBBIs, sexually transmitted and blood‐borne infections.

*p*<0.1+; *p*≤0.05*, *p*≤0.01**, *p*≤0.001***.

Specifically, monetary incentives significantly improved self‐reported adherence (>90%) (OR = 5.9, 1.9−18.2, *p*<0.01) [56], although such effects weakened over time [57]. Similarly, lottery incentives linked to 90% adherence significantly increased adherence over 9 months (adjusted β = 24.8, 4.0−45.7, *p*<0.05) [58]. However, lottery incentives did not significantly improve mean ART adherence over 20 months or viral suppression [59]. In comparison, participants receiving USD 4.50 and USD 10 incentives demonstrated significantly higher viral suppression rates (<1000 copies/mL) at 6 months among those retained in care (RD = 9.8, 1.2−18.5, *p*<0.05 and RD = 13.0, 4.5−21.5, *p*<0.01, respectively) [60]. Another study reported significantly higher viral suppression rates with monetary incentives (OR = 14.3, 4.3−47.7, *p*<0.01) [56]. However, monetary incentives targeting provider visits did not significantly improve viral suppression at either the fifth or sixth visit [34]. Taken together, these findings suggest that incentive‐based behavioural economics‐informed interventions, particularly monetary incentives, showed generally positive effects on STBBI treatment adherence outcomes, especially for retention in care and ART adherence among PLWH, while findings for lottery incentives and viral suppression were less consistent across studies.

### 3.6 Risk of Bias Assessment

Our risk‐of‐bias assessment identified considerable variability in the methodological quality across the included studies (Appendix S3 and Figure S3). Among individually randomized trials, the randomization process and outcome measurement were often judged to be of low risk, whereas concerns frequently arose from deviations from the intended interventions, missing outcome data and the selection of the reported result. Cluster RCTs showed a similar pattern, with additional concerns related to the randomization process and the timing of participant identification or recruitment. Overall, 27 of 40 included RCTs (67.5%) were judged to have some concerns or a high risk of bias. Table S2 (Appendix S4) summarizes the direction of effect, overall RoB judgement, sample size and follow‐up duration for each included RCT.

## 4 Discussion

This systematic review provides a cross‐STBBI synthesis of behavioural economics‐informed interventions across prevention, care engagement and treatment adherence. It moves beyond a single‐disease focus by examining how behavioural economics‐informed strategies are applied across comparable stages of the care cascade in different STBBI contexts. This broader perspective enables comparison of intervention evidence across diseases and cascade stages, helping identify shared patterns and stage‐specific differences. Our findings suggest that monetary incentives showed the clearest promise for prevention, whereas evidence for crowdsourcing strategies was less consistent. In care engagement, diverse strategies such as defaults, incentives and pay‐it‐forward models improved testing uptake, though they showed limited success in linkage to care. For treatment adherence, where the evidence was primarily HIV‐related, incentives predominated and showed positive effects on retention, ART adherence and viral suppression, despite some inconsistencies. This stage‐based synthesis provides useful insights for the design of future behavioural interventions to strengthen STBBI prevention and care.

Incentive‐based strategies, particularly monetary and voucher types, were frequently employed and effective across all three stages of the STBBI care cascade [14, 15]. Within STBBI prevention, monetary incentives promoted safer sex [42], while both voucher and monetary incentives increased male circumcision [43], with fixed compensation outperforming lotteries [45]. Lotteries showed some benefits for condom use [43]. In STBBI care engagement, monetary incentives consistently enhanced HIV testing [28, 33, 47, 61], and vouchers were effective for HIV self‐testing [29], and for chlamydia screening kit returns [32], outperforming lotteries in the latter. Also, in STBBI treatment adherence, monetary incentives significantly improved retention [55], ART adherence [56], the effects of which sometimes waned [57], and viral suppression [55, 56]. Lotteries showed mixed results for ART adherence and viral suppression [58, 59]. These findings suggest a hierarchy of effectiveness linked to the intensity and value of rewards. Optimizing incentives across the cascade requires considering their intensity and timing; higher‐value [42, 44, 60] and immediate rewards [28] often yield better results. Taken together, incentive‐based interventions appeared particularly relevant for testing uptake within care engagement and for some treatment‐adherence outcomes, with monetary incentives generally showing more consistent effects than lottery‐based approaches. One possible explanation is that monetary incentives may help offset immediate costs and reduce hesitation or inertia surrounding action, while also supporting continued engagement with treatment‐related behaviours beyond initial uptake [61, 62].

Beyond direct incentives, other behavioural economic strategies were more commonly applied at the STBBI care engagement stage. Defaults, particularly opt‐out and active‐choice approaches for HIV testing and HCV screening [27, 30, 47], demonstrated strong, sustained and scalable effects. Framing interventions showed more context‐dependent utility, improving HIV testing when gain‐framed [31] but showing limited benefit for linkage to care [10]. Norm‐based interventions yielded mixed results for HCV screening [30] and ART adherence [63]. Simple commitment strategies were largely ineffective for HIV testing [28, 33, 61]. By contrast, combined strategies, such as planning prompts with reminders for HIV kit returns [35], were more effective than standalone approaches like planning prompts alone for HIV testing uptake [36]. This pattern may reflect the nature of care engagement behaviours, which often involve discrete actions embedded in service delivery processes, such as accepting a test, completing screening or returning a kit. In such contexts, strategies that alter the choice environment, reduce friction or prompt action at the point of decision may be more effective than approaches that rely solely on intention or message framing. The stronger performance of combined approaches further suggests that behaviours at this stage are often shaped by multiple behavioural bottlenecks rather than a single barrier.

Innovative behavioural economic approaches also primarily targeted STBBI care engagement, particularly testing. Pay‐it‐forward strategies, leveraging reciprocity, successfully boosted dual chlamydia/gonorrhoea and HBV/HCV testing [38, 39, 48], often proving cost‐effective or cost‐saving and offering a sustainable alternative. This may be because pay‐it‐forward approaches reduce financial barriers while increasing the social meaning and acceptability of testing by framing participation as a shared and prosocial act. Crowdsourcing was primarily used to develop culturally resonant health materials and support interventions throughout the cascade. However, its direct impact on behaviour change within the engagement stage was inconsistent [40, 46, 49, 50, 51, 52]. One possible explanation is that crowdsourcing mainly improves intervention acceptability, message relevance and cultural resonance, while leaving other barriers to action unresolved. In this sense, crowdsourcing may function more as an intervention design enabler than as a strong standalone lever for behaviour change.

An additional consideration is the heterogeneity of intervention effects across studies. Prevention‐related behaviours such as MMC often involve a one‐off or intermittent decision [44, 45], whereas treatment adherence requires repeated action and sustained engagement over time [53]. These differences suggest that the dominant behavioural barriers and the intervention features needed to address them vary across cascade stages and may partly explain differences in intervention durability. Effects were generally clearer over short or medium follow‐up periods, whereas longer‐term durability was less consistently demonstrated. For example, lottery incentives improved condom use at 3 months but not at 6 months [43], and one monetary incentive study for ART adherence showed attenuation over time [57]. Short‐term behaviour change may, therefore, be sufficient for discrete actions such as testing uptake or MMC, whereas HIV treatment adherence requires effects that can be sustained over time. This distinction may also differ across STBBIs, as some infections involve shorter treatment windows or curable episodes, whereas HIV requires ongoing long‐term management. Responses may also vary across populations and settings, shaped by stigma, financial constraints, perceived risk, prior engagement with care, cultural norms and service accessibility. For socially mediated approaches such as pay‐it‐forward [39, 48] and crowdsourcing [25, 40], local norms around reciprocity, trust and community engagement may be especially relevant [12]. These findings suggest that behavioural economics‐informed interventions are context‐dependent and may work best when matched to the behavioural and structural barriers most relevant to a given population and stage of the care cascade.

This review has several limitations. First, by excluding conference abstracts, which often lack sufficient detail for a full synthesis and quality appraisal, we may have missed some recent findings. Second, substantial heterogeneity across populations, interventions, diseases and outcomes precluded meta‐analysis and limited direct comparisons across studies. In addition, the included STBBIs differ in stigma, clinical course, treatment burden and care pathways, which may affect both behavioural barriers and intervention effects. The evidence base was also uneven across diseases and care cascade stages, with HIV‐related outcomes reported in 29 of the 40 included RCTs. Evidence for treatment adherence was concentrated largely in HIV, which limits generalizability across all STBBIs. Third, the strength of the evidence is constrained by the methodological quality of the included trials; most studies had some concerns or a high risk of bias, primarily from deviations from the intended intervention and selective reporting. As shown in Table S2, several favourable findings were identified in studies with concerns or high risk of bias, short follow‐up periods or modest sample sizes, which should be taken into account when assessing the robustness and transferability of the evidence. Finally, our reliance on published literature introduces the risk of publication bias, as studies with positive findings are more likely to be published, potentially leading to an overestimation of intervention effects and implications for policy and practice.

These findings across the STBBI care cascade have several key implications for public health. Programme design can be enhanced by strategically selecting interventions, such as defaults for testing, pay‐it‐forward for specific populations and tailored incentives based on their effectiveness at different stages of the cascade. Resource allocation towards cost‐effective behavioural strategies for each stage necessitates routine health economic analyses. While tailored interventions can improve equity, careful design is essential. Context‐specific intervention design should reflect the duration and behavioural demands of the target outcome. Discrete or time‐limited actions may benefit from interventions that generate immediate behaviour change, whereas chronic care behaviours require effects that can be sustained over time. In the present review, evidence on the treatment adherence stage was limited to HIV‐related outcomes, highlighting an important evidence gap regarding the long‐term effectiveness of behavioural economics‐informed strategies in other STBBI contexts involving longer‐term management needs. Sustainability can be improved by shifting from time‐limited incentives to durable nudges, such as defaults, and by exploring inherently sustainable approaches, such as pay‐it‐forward. Ultimately, realizing these benefits requires building public health workforce capacity to design, implement and evaluate these nuanced, cascade‐stage‐specific interventions.

## 5 Conclusions

In conclusion, behavioural economics‐informed interventions hold substantial promise for optimizing the STBBI care cascade. Incentives and defaults showed the most consistent benefit, although their effects remained sensitive to intervention design and implementation context. Sustainable alternatives such as pay‐it‐forward also showed promise for improving screening. However, the evidence base remained uneven across diseases and stages of the care cascade. No single strategy was universally optimal, and the effectiveness of approaches such as framing, norms and crowdsourcing varied across settings. Future research should build on existing evidence on behavioural economics‐informed interventions in STBBIs and assess their transferability across diseases in context‐specific settings to inform wider implementation and maximize global public health impact.

## Author Contributions

MZ led the conceptualization and design of the study, performed screening and study selection, quality assessment, data analysis and synthesis, and wrote the initial draft of the manuscript. ZT contributed to the literature search, data extraction, evaluation and drafting of the Methods section. XZ contributed to the creation of the figure and the revision of the manuscript. JJO, WT, YZ, JS and CL critically revised the manuscript for important intellectual content. FJ and LZ contributed to the study's conceptualization and design, provided project administration and supervision, and critically revised the manuscript. All authors have read and approved the final manuscript.

## Funding

The study was supported by the Ministry of Education in China Project of Humanities and Social Sciences (24YJC840051), the Natural Science Foundation of Shaanxi Province (2025JC‐YBQN‐1106), the Fundamental Research Funds for the Central Universities (SK2026027), the Ministry of Science and Technology of the People's Republic of China (2022YFC2304900, 2022YFC2304905) and the National Key R&D Programme of China (2022YFC2505100, 2022YFC2505103).

## Conflicts of Interest

The authors declare that they have no conflicts of interest.

## Supporting information




**Supporting File 1**: jia270136‐sup‐0001‐SuppMat.docx

## Data Availability

All data generated or analysed during this study are included in this published article (and its Supplementary Information Files). The datasets are derived from publicly available resources listed in the references.
