# Supplementary material for: The Impact of Behavioural Economics‐Informed Interventions on the Care Cascade for Sexually Transmitted and Blood‐Borne Infections: A Systematic Review of Randomized Controlled Trials
Source: J Int AIDS Soc. 2026 Aug 10;29(8):e70136. doi: 10.1002/jia2.70136 (PMC13458029; doi:10.1002/jia2.70136)
Supplement: Supplementary file 1 [file JIA2-29-e70136-s001.docx]

# Appendix

**Table of Contents**

[Appendix 1: PRISMA 2020 Checklist	2](#_Toc225929307)

[Appendix 2: Search Strategy	5](#_Toc225929308)

[Appendix 3: Supplementary figures	8](#_Toc225929309)

[Appendix 4: Supplementary tables	12](#_Toc225929310)

[Reference	25](#_Toc225929311)

## Appendix 1: PRISMA 2020 Checklist

| **Section and Topic** | **Item #** | **Checklist item** | **Location where item is reported** |
| --- | --- | --- | --- |
| **TITLE** | | |  |
| Title | 1 | Identify the report as a systematic review. | p 1 |
| **ABSTRACT** | | |  |
| Abstract | 2 | See the PRISMA 2020 for Abstracts checklist. |  |
| **INTRODUCTION** | | |  |
| Rationale | 3 | Describe the rationale for the review in the context of existing knowledge. | pp 3-4 |
| Objectives | 4 | Provide an explicit statement of the objective(s) or question(s) the review addresses. | p 5 |
| **METHODS** | | |  |
| Eligibility criteria | 5 | Specify the inclusion and exclusion criteria for the review and how studies were grouped for the syntheses. | pp 6-7 |
| Information sources | 6 | Specify all databases, registers, websites, organisations, reference lists and other sources searched or consulted to identify studies. Specify the date when each source was last searched or consulted. | p 6 |
| Search strategy | 7 | Present the full search strategies for all databases, registers and websites, including any filters and limits used. | Appendix 2, pp 5-7 |
| Selection process | 8 | Specify the methods used to decide whether a study met the inclusion criteria of the review, including how many reviewers screened each record and each report retrieved, whether they worked independently, and if applicable, details of automation tools used in the process. | pp 7 |
| Data collection process | 9 | Specify the methods used to collect data from reports, including how many reviewers collected data from each report, whether they worked independently, any processes for obtaining or confirming data from study investigators, and if applicable, details of automation tools used in the process. | pp 7 |
| Data items | 10a | List and define all outcomes for which data were sought. Specify whether all results that were compatible with each outcome domain in each study were sought (e.g. for all measures, time points, analyses), and if not, the methods used to decide which results to collect. | p 7 |
|  | 10b | List and define all other variables for which data were sought (e.g. participant and intervention characteristics, funding sources). Describe any assumptions made about any missing or unclear information. | pp 6-7 |
| Study risk of bias assessment | 11 | Specify the methods used to assess risk of bias in the included studies, including details of the tool(s) used, how many reviewers assessed each study and whether they worked independently, and if applicable, details of automation tools used in the process. | p 9 |
| Effect measures | 12 | Specify for each outcome the effect measure(s) (e.g. risk ratio, mean difference) used in the synthesis or presentation of results. | pp 7-8 |
| Synthesis methods | 13a | Describe the processes used to decide which studies were eligible for each synthesis (e.g. tabulating the study intervention characteristics and comparing against the planned groups for each synthesis (item #5)). | p 9 |
|  | 13b | Describe any methods required to prepare the data for presentation or synthesis, such as handling of missing summary statistics, or data conversions. | p 9 |
|  | 13c | Describe any methods used to tabulate or visually display results of individual studies and syntheses. | p 9 |
|  | 13d | Describe any methods used to synthesize results and provide a rationale for the choice(s). If meta-analysis was performed, describe the model(s), method(s) to identify the presence and extent of statistical heterogeneity, and software package(s) used. | p 9 |
|  | 13e | Describe any methods used to explore possible causes of heterogeneity among study results (e.g. subgroup analysis, meta-regression). | NA |
|  | 13f | Describe any sensitivity analyses conducted to assess robustness of the synthesized results. | NA |
| Reporting bias assessment | 14 | Describe any methods used to assess risk of bias due to missing results in a synthesis (arising from reporting biases). | NA |
| Certainty assessment | 15 | Describe any methods used to assess certainty (or confidence) in the body of evidence for an outcome. | p 9 |
| **RESULTS** | | |  |
| Study selection | 16a | Describe the results of the search and selection process, from the number of records identified in the search to the number of studies included in the review, ideally using a flow diagram. | p 10 |
|  | 16b | Cite studies that might appear to meet the inclusion criteria, but which were excluded, and explain why they were excluded. | NA |
| Study characteristics | 17 | Cite each included study and present its characteristics. | p 10; Appendix 4, Table S1 |
| Risk of bias in studies | 18 | Present assessments of risk of bias for each included study. | p 16; Appendix 4, pp 10-11; PP15-23 |
| Results of individual studies | 19 | For all outcomes, present, for each study: (a) summary statistics for each group (where appropriate) and (b) an effect estimate and its precision (e.g. confidence/credible interval), ideally using structured tables or plots. | pp 10-13; Table 1-Table 3 |
| Results of syntheses | 20a | For each synthesis, briefly summarise the characteristics and risk of bias among contributing studies. | pp 10-13 |
|  | 20b | Present results of all statistical syntheses conducted. If meta-analysis was done, present for each the summary estimate and its precision (e.g. confidence/credible interval) and measures of statistical heterogeneity. If comparing groups, describe the direction of the effect. | pp 10-13; Table 1-Table 3 |
|  | 20c | Present results of all investigations of possible causes of heterogeneity among study results. | Appendix 4, Table S2 |
|  | 20d | Present results of all sensitivity analyses conducted to assess the robustness of the synthesized results. | Appendix 4, Table S2 |
| Reporting biases | 21 | Present assessments of risk of bias due to missing results (arising from reporting biases) for each synthesis assessed. | Appendix 3, Figure S3a; FigureS3b |
| Certainty of evidence | 22 | Present assessments of certainty (or confidence) in the body of evidence for each outcome assessed. | Appendix 4, Table S2 |
| **DISCUSSION** | | |  |
| Discussion | 23a | Provide a general interpretation of the results in the context of other evidence. | p 15 |
|  | 23b | Discuss any limitations of the evidence included in the review. | pp 17-18 |
|  | 23c | Discuss any limitations of the review processes used. | p 17 |
|  | 23d | Discuss implications of the results for practice, policy, and future research. | P 18 |
| **OTHER INFORMATION** | | |  |
| Registration and protocol | 24a | Provide registration information for the review, including register name and registration number, or state that the review was not registered. | p 2; p6 |
|  | 24b | Indicate where the review protocol can be accessed, or state that a protocol was not prepared. | p 6 |
|  | 24c | Describe and explain any amendments to information provided at registration or in the protocol. | NA |
| Support | 25 | Describe sources of financial or non-financial support for the review, and the role of the funders or sponsors in the review. | p 20 |
| Competing interests | 26 | Declare any competing interests of review authors. | p 20 |
| Availability of data, code and other materials | 27 | Report which of the following are publicly available and where they can be found: template data collection forms; data extracted from included studies; data used for all analyses; analytic code; any other materials used in the review. | Supplementary file, appendix1- appeddix4 |

*From:* Page MJ, McKenzie JE, Bossuyt PM, Boutron I, Hoffmann TC, Mulrow CD, et al. The PRISMA 2020 statement: an updated guideline for reporting systematic reviews. BMJ 2021;372:n71. doi: 10.1136/bmj.n71

## Appendix 2: Search Strategy

A comprehensive search was conducted across five major databases: MEDLINE (via PubMed), Embase, CINAHL (via EBSCOhost), Web of Science (Core Collection), and the Cochrane Central Register of Controlled Trials (CENTRAL). The search was performed on April 25, 2024, with no restrictions on language, publication date, or geographical setting. To capture recently published studies, an updated database search was conducted prior to the final analysis on March 22, 2025.

**PubMed**

(((( "Acquired Immunodeficiency Syndrome"[Mesh] OR "Acquired Immunodeficiency Syndrome"[All Fields] OR AIDS[All Fields] OR HIV[Mesh] OR HIV[All Fields] OR "Sexually Transmitted Diseases"[Mesh] OR "Sexually Transmitted Disease*"[All Fields] OR STD[All Fields] OR STDs[All Fields] OR "Human Papillomavirus Viruses"[Mesh] OR "Human Papillomavirus Virus*"[All Fields] OR HPV[All Fields] OR "Herpes Simplex"[Mesh] OR "Herpes Genitalis"[Mesh] OR HSV[All Fields] OR "Herpes Simplex Virus"[All Fields] OR "Genital Herpes"[All Fields] OR "Hepatitis B Virus"[Mesh] OR "Hepatitis B Virus"[All Fields] OR HBV[All Fields] OR "Hepacivirus"[Mesh] OR "Hepacivirus"[All Fields] OR HCV[All Fields] OR "chronic viral hepatitis"[All Fields] OR "Chlamydia Infections"[Mesh] OR Chlamydia[All Fields] OR "Chlamydia trachomatis"[All Fields] OR "Gonorrhea"[Mesh] OR Gonorrhea[All Fields] OR "Neisseria gonorrhoeae"[All Fields] OR "Syphilis"[Mesh] OR Syphilis[All Fields] OR "Treponema pallidum"[All Fields] OR STI[All Fields] OR STIs[All Fields] OR "Sexually Transmitted infection*"[All Fields] ))

AND

(("Economics, Behavioral"[Mesh]) OR ("behavioral economic*" [All Fields]) OR (nudg* [All Fields]) OR (Choice architecture [All Fields]) OR ("behavioral control" [All Fields]) OR ("behavioral intervention*"[All Fields]) OR ("behavioral strateg*" [All Fields]) OR (behavioral insight* [All Fields]) OR ("behavioural economic*" [All Fields]) OR ("behavioural control" [All Fields]) OR ("behavioural intervention"[All Fields]) OR ("behavioural strateg*" [All Fields]) OR ("behavioural insight*" [All Fields]) OR ("pay-it-forward" [All Fields]) OR ("pay it forward" [All Fields]) OR (crowdsourc* [All Fields]) OR (nudgeathon* [All Fields])))

AND

("randomised controlled trial*"[Title/Abstract] OR "randomized controlled trial*"[Title/Abstract] OR "RCTs"[Title/Abstract] OR "RCT"[Title/Abstract]))

NOT

("Review"[Title] OR "systematic review"[Title] OR "meta-analysis"[Title] OR "scoping review"[Title] OR "umbrella review"[Title] OR "protocol"[Title])

**Embase**

**#1** 'human immunodeficiency virus'/exp OR 'acquired immune deficiency syndrome'/exp OR 'sexually transmitted disease'/exp OR 'human papillomavirus'/exp OR 'hepatitis b'/exp OR 'hepatitis c'/exp OR 'chlamydia trachomatis'/exp OR 'gonorrhea'/exp OR 'syphilis'/exp OR 'herpes simplex'/exp OR 'genital herpes'/exp OR 'pre-exposure prophylaxis'/exp OR 'post exposure prophylaxis'/exp OR 'antiretroviral therapy'/exp

**#2** 'human immunodeficiency virus' OR hiv OR 'acquired immune deficiency syndrome' OR aids OR 'sexually transmitted disease' OR std OR stds OR sti OR stis OR 'human papillomavirus' OR hpv OR 'wart virus' OR 'hepatitis b' OR hbv OR 'hepatitis c' OR hcv OR 'chronic viral hepatitis' OR chlamydia OR 'chlamydia trachomatis' OR gonorrhea OR 'neisseria gonorrhoeae' OR syphilis OR 'treponema pallidum' OR 'herpes simplex virus' OR hsv OR 'genital herpes' OR 'pre-exposure prophylaxis' OR prep OR 'post-exposure prophylaxis' OR pep OR 'antiretroviral therapy' OR art.

**#3** #1 OR #2

**#4** ‘behavioral economics’/exp OR ‘behavioral economic*’ OR nudg* OR ‘Choice architecture’ OR 'behavioral control' OR 'behavioral intervention*' OR 'behavioral strateg*' OR 'behavioral insight*' OR 'behavioural economic*' OR 'behavioural control' OR 'behavioural intervention*' OR 'behavioural strateg*' OR 'behavioural insight*' OR 'pay-it-forward' OR 'pay it forward' OR crowdsourc* OR nudgeathon*

**#5** 'randomised controlled trial*':ti,ab,kw OR 'randomized controlled trial*':ti,ab,kw OR 'rcts':ti,ab,kw OR 'rct':ti,ab,kw

**#6** #3 AND #4 AND #5 NOT (review:ti OR 'systematic review':ti OR 'meta-analysis':ti OR 'scoping review':ti OR 'umbrella review':ti OR protocol:ti)

**CINAHL(EBSCOhost)**

S1 ( (DE "AIDS" OR DE "HIV" OR DE "SEXUALLY transmitted diseases" OR DE "HUMAN papillomavirus" OR DE "HEPATITIS B virus" OR DE "HEPATITIS C virus" OR DE "CHLAMYDIA" OR DE "GONORRHEA" OR DE "SYPHILIS" OR DE "HERPES SIMPLEX" OR DE "GENITAL HERPES") OR TX ( "Acquired Immunodeficiency Syndrome" OR AIDS OR "Human immunodeficiency virus*" OR HIV OR "Sexually Transmitted Disease*" OR STD OR STDs OR STI OR STIs OR "Human Papillomavirus*" OR HPV OR "Herpes Simplex Virus*" OR HSV OR "Genital Herpes" OR "Hepatitis B Virus*" OR HBV OR "Hepatitis C Virus*" OR HCV OR "chronic viral hepatitis" OR Chlamydia OR "Chlamydia trachomatis" OR Gonorrhea OR "Neisseria gonorrhoeae" OR Syphilis OR "Treponema pallidum" OR "Pre-Exposure Prophylaxis" OR "PrEP" OR "Post-Exposure Prophylaxis" OR "PEP" OR "Antiretroviral Therapy" OR "ART" ) )

S2 (DE "BEHAVIORAL economics") OR TX ("behavioral economic*" OR nudg* OR "Choice architecture" OR "behavioral control" OR "behavioral intervention*" OR "behavioral strateg*" OR "behavioral insight*" OR "behavioural economic*" OR "behavioural control" OR "behavioral intervention*" OR "behavioral strateg*" OR "behavioral insight*" OR "pay-it-forward" OR "pay it forward" OR crowdsourc* OR nudgeathon*)

S3 TI ( "Randomised Controlled Trial*" OR "Randomized Controlled Trial*" OR "RCTs" OR "RCT" ) OR AB ( "Randomised Controlled Trial*" OR "Randomized Controlled Trial*" OR "RCTs" OR "RCT") OR KW ("Randomised Controlled Trial*" OR "Randomized Controlled Trial*" OR "RCTs" OR "RCT" )

S4 TI (Review OR "systematic review" OR "meta-analysis" OR "scoping review" OR "umbrella review" OR protocol)

S5 (S1 AND S2 AND S3) NOT S4

**Web of science**

**1:** ALL=("human immunodeficiency virus*" OR HIV OR "acquired immunodeficiency syndrome" OR AIDS OR "human papillomavirus*" OR HPV OR "herpes simplex virus*" OR HSV OR "genital herpes" OR "hepatitis b virus*" OR HBV OR "hepatitis c virus*" OR HCV OR "chronic viral hepatitis" OR chlamydia OR "chlamydia trachomatis" OR gonorrhea OR "neisseria gonorrhoeae" OR syphilis OR "treponema pallidum" OR "sexually transmitted disease*" OR STD OR STDs OR "sexually transmitted infection*" OR STI OR STIs OR "pre-exposure prophylaxis" OR "pre exposure prophylaxis" OR PrEP OR "post-exposure prophylaxis" OR "post exposure prophylaxis" OR PEP OR "antiretroviral therapy" OR ART)

**2:** ALL=("behavioral economic*" OR nudg* OR "choice architecture*" OR "behavioral control" OR "behavioral intervention*" OR "behavioral strateg*" OR "behavioral insight*" OR "behavioural economic*" OR "behavioural control" OR "behavioural intervention*" OR "behavioural strateg*" OR "behavioural insight*" OR "pay-it-forward" OR "pay it forward" OR crowdsourc* OR nudgeathon*)

**3:** ((TI=("randomised controlled trial*" OR "randomized controlled trial*" OR "rcts" OR "rct" )) OR AB=("randomised controlled trial*" OR "randomized controlled trial*" OR "rcts" OR "rct" )) OR KP=("randomised controlled trial*" OR "randomized controlled trial*" OR "rcts" OR "rct" )

**4:** #1 AND #2 AND #3

**5:** TI=(review OR "systematic review" OR "meta-analysis" OR "scoping review" OR "umbrella review" OR protocol)

**6:** (#4) NOT #5

**Cochrane**

#1 (((human immunodeficiency virus* OR HIV OR acquired immunodeficiency syndrome OR AIDS OR human papillomavirus* OR HPV OR herpes simplex virus* OR HSV OR genital herpes OR hepatitis b virus* OR HBV OR hepatitis c virus* OR HCV OR chronic viral hepatitis) OR (chlamydia OR chlamydia trachomatis OR gonorrhea OR neisseria gonorrhoeae OR syphilis OR treponema pallidum) OR (sexually transmitted disease* OR STD OR STDs OR sexually transmitted infection* OR STI OR STIs) OR (pre-exposure prophylaxis OR pre exposure prophylaxis OR PrEP OR post-exposure prophylaxis OR post exposure prophylaxis OR PEP OR antiretroviral therapy OR ART)))

#2 ((behavioral NEXT economic*) OR nudg* OR (choice NEXT architecture*) OR

(behavioral NEXT control) OR (behavioral NEXT intervention*) OR

(behavioral NEXT strateg*) OR (behavioral NEXT insight*) OR

(behavioural NEXT economic*) OR (behavioural NEXT control) OR

(behavioural NEXT intervention*) OR (behavioural NEXT strateg*) OR

(behavioural NEXT insight*) OR ("pay-it-forward") OR ("pay it forward") OR

crowdsourc* OR nudgeathon*)

#3 ((randomised controlled trial*) OR (randomized controlled trial*) OR rcts OR rct):ti,ab,kw

#4 (protocol:ti OR review:ti)

#5 (#1 AND #2 AND #3) NOT #4

## Appendix 3: Supplementary figures

**Previous reviews**

**Identification of update studies via databases**

**Identification of studies via databases and registers**

Records excluded:

Irrelevant (n =4)

Non-RCTs (eg. review, qualitative, cross-sectional study, protocol (n= 7)

Records removed *before screening*:

Duplicate records removed

(n = 63)

Additional records identified through updated database (N=75)†

PubMed (n=30)

Embase (n=25)

Web of Science (n=20)

Records identified from

Databases (N = 2461)*

PubMed (n=230)

Embase (n=336)

Web of Science (n=551)

Cochrane (n=727)

CINAHL (n=617)

Records identified from: Citation searching

(n = 10)

**Identification**

Records removed *before screening*:

Duplicate records removed

(n = 63)

Records removed *before screening*:

Duplicate records removed

(n = 1042)

Records screened

(n =12)

Records screened

(n =1419)

**Screening**

Records excluded:

Irrelevant (n =4)

Non-RCTs (eg. review, qualitative, cross-sectional study, protocol (n= 7)

Records excluded:

Duplicate (n=12)

Irrelevant (n =1029)

Non-RCTs (eg. review, qualitative, cross-sectional study, protocol (n= 317)

Conference abstract (n = 32)

Eligible Records

(n = 1)

Eligible Records

(n = 29)

**Included**

Studies included in review

(n = 40)

**Figure S1. Study selection.** PRISMA flow diagram depicting the selection process for publications included in this systematic review. *Search in April 25 2024. †Updated search in March 2025.

**Figure S2. Distribution of behavioural economics-informed interventions and their countries of implementation in 40 RCTs.**

**Figure S3a. Risk of bias results of individual RCTs (N=34)**

**Figure S3b. Risk of bias results of cluster RCTs (N=6)**

## Appendix 4: Supplementary tables

**Table S1. Behavioral economic strategies and applications in included RCTs.**

| Strategies | Grounds | Behavioral Economic-informed Interventions in HIV/STD Care |
| --- | --- | --- |
| Incentives (Monetary) | Monetary incentives help overcome structural, interpersonal, and individual barriers, such as cost and stigma, to promote healthy behaviors. They provide the necessary motivation to initiate action by countering inertia or hesitation^.^.^1,2^ | 1. Three monetary incentive groups: $1, $5, and $10 offered for HIV testing.^3^ 2. Two groups received conditional cash transfers for negative STI tests: low-value ($10 per round) or high-value ($20 per round).^4^ 3. Participants received 25,000 USh (approximately US$7 in 2018) for HIV retesting at 3 and 6 months.^5^ 4. Participants received $10 for HIV testing to cover time and transportation costs.^2^ 5. Two intervention groups were offered $10 for HIV testing: one received the incentive immediately upon testing, the other upon receiving results three weeks later.^6^ 6. Participants received $30 for each attended provider visit.^7^ 7. Two groups received standard care plus monthly cash incentives: 10,000 TZS (≈$4.50) or 22,500 TZS (≈$10.00) for clinic attendance.^8^ 8. Participants received $5 plus $1 per visit for attending prevention of mother-to-child transmission of HIV (PMTCT) appointments.^9^ 9. Participants earned up to $10 daily for two years for providing blood samples with reduced or undetectable viral loads.^10^ 10. Participants received escalating financial incentives ($4 to $12.50) for viral suppression at 6, 12, and 24 weeks.^11^ 11. Participants received both daily text message reminders and conditional incentives ($0.15–$3 USD) tied to their pill-taking behavior.^12^ |
| Incentives  (Lottery) | Optimism bias leads individuals to overestimate their chances of winning, making lottery-based incentives effective despite low statistical probabilities.^13^ | 1. Participants could enter a lottery for circumcision, with prizes ranging from a US $2.50 food voucher to a US $120 bicycle, and varying probabilities of winning.^14^ 2. A lottery using scratch cards with odds from 1:10 to 1:25 offered prizes like phones and bikes for HIV testing completion.^15^ 3. Participants were offered the chance to participate in a £200 HMV voucher lottery if they picked up the screening test and returned it.^16^ 4. Participants received transport compensation and a 50% chance to win R400(≈US$33) to improve study visit attendance.^17^ 5. Intervention participants were entered into a daily lottery for $10 or $100 if one or two digits matched after opening their electronic pill bottle.^18^ 6. Participants could win lottery prizes for attending appointments, achieving viral suppression, and demonstrating high medication adherence via MEMS-cap data.^19^ 7. Participants were incentivized with lottery drawings for attending appointments on time and achieving high medication adherence.^20,21^ 8. To encourage HIV care continuum engagement, participants could win a mobile phone, data, or a R1000(≈$100) gift card through lotteries at key milestone.^22^ |
| Incentives  (voucher) | People tend to be more motivated by avoiding losses than gaining equivalent gains, and often value possessions more highly than items they don't yet own ^23^. | 1. Participants randomized to the 3 intervention groups received food vouchers worth 200, 700, or 1200 Kenya shillings (≈US $2.50, $8.75, or $15.00) upon undergoing voluntary medical male circumcision.^24^ 2. The Malkia Klabu loyalty program offers a non-cash incentive, rewarding participants with a points system or vouchers, to encourage young women to access HIV self-testing and contraceptive services at drug shops.^25^ 3. A voucher-like brochure, resembling a ZAR 100 (US$7) HIV test voucher, promoted HIV testing at participating clinics.^23^ 4. Participants could earn or lose a £5 HMV voucher based on their engagement with return Chlamydia screening kits.^16^ 5. The intervention group received a R300 (∼US $23) cash voucher redeemable upon initiating ART within 3 months.^26^ |
| Defaults | Defaults leverage status quo bias by pre-selecting choices, assuming individuals are more likely to stick with pre-set options unless they actively changed ^1^. | 1. Patients were offered HIV testing through opt-in, active-choice, and opt-out approaches.^3,27^ 2. Participants received a letter and lab requisition form for HCV antibody screening, choosing to opt-in or opt-out by submitting the form.^28^ |
| Framing | Framing shapes decision-making by presenting information in specific contexts, making certain choices appear more appealing through comparison or association.^1^ | 1. Participants in the loss-framed group chose a prize and were told they would lose it if they didn't get tested for HIV.^15^ 2. The peer-delivered intervention promoted U=U, emphasizing that HIV treatment enables PLHIV to live healthy, normal lives and prevent transmission.^29^ 3. To reduce HIV testing stigma, a brochure bundled free HIV tests with other health screenings (blood pressure, glucose, BMI), simplifying decision-making.^23^ 4. Participants were incentivized to return Chlamydia screening kits with gain-framed (voucher/lottery) or loss-framed (voucher/lottery) approaches. ^16^ |
| Commitment | Commitment strategies utilize pre-commitment to counter present-bias, helping individuals align current actions with long-term goals.^30^ | 1. Participants could voluntarily deposit 20,000 Ush (≈US$6) to commit to retesting in 3 months, earning 5,000 Ush (≈US$1) interest upon return.^5^ 2. Participants could choose a commitment contract, receiving $30 per visit for attending and demonstrating 90% ART adherence.^7^ 3. Participants made a soft-commitment to get tested within 15 days by signing a form and receiving HIV testing information.^2^ 4. Participants made a soft-commitment to HIV testing within two weeks by choosing a statement and initialing it.^6^ |
| Norms | Social norms significantly influence behavior, as individuals often align their actions with observed peer behaviors or prevalent social trends.^31^ | 1. Participants received feedback on their own adherence and the adherence of their peers.^32^ 2. Text messages used social norms, anticipated regret, reciprocity, and commitment to encourage HCV screening.^28^ |
| Planning  prompts | Planning prompts encourage individuals to detail future actions, linking intention with implementation, reducing procrastination and forgetfulness.^30^ | 1. A planning prompt encouraged participants to schedule time for HIV testing and return the kit, boosting self-efficacy.^33^ 2. Participants were prompted to schedule a specific date and time for their HIV test on a calendar, enhancing planning.^34^ |
| Reminders | Reminders reduce the cognitive load of completing tasks by drawing attention to future consequences and triggering action through memory cues. ^35^ | 1. Assist with setting automated prompts to seek HPV vaccinations once medically eligible post-pregnancy. ^36^ 2. Two reminder messages were sent: one leveraging reciprocity and the other prompting immediate action with a deadline and HIV kit expiry date. ^33^ |
| Salience | People's behaviour is greatly influenced by what their attention is drawn to. By increasing the salience of certain information or behaviors, individuals' choices and actions can be effectively influenced. ^37^ | 1. Participants received daily text message reminders for 3 months to keep adherence and their anchoring strategy salient.^12^ |
| Pay-it-forward | Pay-it-forward fits within the broader field of behavioral economics.^38^ Pay-it-forward involves generalized reciprocity, where kindness received prompts subsequent pro-social behavior towards unrelated others, often driven by affective responses like gratitude or warm glow. ^39^ | 1. Participants were told the standard testing price was ¥150 (US$22) and received a free test funded by prior participants' donations. They could then voluntarily choose whether and how much to donate towards future testing for others. ^40^  2. Participants were informed the standard testing price was ¥150 (US$22) and received free testing. They then decided the amount they would like to pay for their own test. ^40^  3. The Pay-It-Forward group received free chlamydia and gonorrhea testing (approximately US $11) as a gift and were asked whether they would like to donate money to another person in the community. ^41^  4. Participants were offered HBV and HCV testing as a gift courtesy of the generosity of previous participants, which cost ¥ 50 (US$ 7.7) or more at hospitals in China. Each participant (regardless of whether they accepted or rejected testing) could choose to donate any amount of money to support other men in the community to receive the same HBV and HCV tests. ^42^ |
| Crowdsourcing | Crowdsourcing effectively utilizes intrinsic behavioral economic motivators like altruism, reciprocity, and social image to contribute to the public good. Concurrently, crowdsourcing platforms themselves serve as valuable tools for testing various behavioral interventions, incentive mechanisms, and decision models. ^43^ | 1. Online crowdsourced intervention promoting HBV/HCV testing through WeChat, including multimedia (images/videos) and participatory components: (1) a multimedia component delivered two images and two videos promoting HBV and HCV testing; (2) a participatory component invited men to compose and submit suggestions for how to better tailor the two images and two videos to MSM in China. ^44^  2. The intervention group will be exposed to crowdsourced intervention materials via an online link. The promotional component, includes two videos and two images promoting HBV and HCV testing, with one material shared each week. ^45^  3. The intervention for the crowdsourcing group involved watching a crowdsourced video that emphasized the protective function of condoms by showing a wall protecting against cartoon viruses. ^46^  4. Participants received standard referral cards, incentives, and crowdsourced HIV partner services (education material, HIVST kit for partners, and referral options). ^47^  5. The participant assigned to the intervention group watched the crowdsourced video and received a text message 3 weeks after survey completion inquiring about HIV test uptake and results. ^48^  6. Participants were exposed to crowdsourced intervention materials, including 25 images promoting HIV testing and four videos promoting safe sex, along with the strategy of providing HIV self-testing services through digital tools. ^49^ |

Abbreviations: HIV, human immunodeficiency virus; HBV, hepatitis B virus; HCV, hepatitis C virus; STI, sexually transmitted infection; MSM, men who have sex with men; PLWH, people living with HIV; ART, antiretroviral therapy; MMC, medical male circumcision.

**Table S2. Study-level Summary of Included RCTs and Their Overall Risk-Of-Bias Judgements**

| Study | STBBI / Condition | Population | Comparisons | Outcome(s) assessed and  effect direction ^a^ | Sample size | Intervention  durations | Overall RoB judgement ^b^ |
| --- | --- | --- | --- | --- | --- | --- | --- |
| Prevention | | | | | | | |
| Walque et al., 2012 | Four STIs ^c^ | Young adults | T1: Incentive (Monetary)- $10  T2: Incentive (Monetary)- $20  C: Standard of Care | **Combined prevalence of four STIs at 12 months** ^c^  T1 vs. C: null ^d^  T2 vs. C: positive | Total: 2399  T1: 660  T2: 615  C: 1124 | 12 months | High risk |
| Galárraga et al., 2018 | condom use | Female adults | T: Incentives (Lottery)  C: Standard of Care | **Condom use at 3 months**  T vs. C: positive  **Condom use at 6 months**  T vs. C: null | Total: 100  T: 50  C: 50 | 6 months | High risk |
| Thirumurthy et al., 2014 | MMC | Uncircumcised male adults | T1: Incentives ($2·50 food voucher)  T2: Incentives ($8·75 food voucher)  T3: Incentives ($15 food voucher)  C: Standard of Care | **Voluntary MMC uptake within 2 months:**  T1 vs. C: null  T2 vs. C: positive  T3 vs. C: positive | Total: 1504  T1: 374  T2: 381  T3: 377  C: 370 | 2 months | Low risk |
| Thirumurthy et al., 2016 | MMC | Uncircumcised male adults | T1: Incentives (Lottery)  T2: Fixed Compensation  C: Standard of Care | **Voluntary MMC uptake within 3 Months**  T1 vs. C: null  T2 vs. C: positive  T1 vs. T2: P for Equality Test: positive | Total: 903  T1: 302  T2: 308  C: 299 | 3 months | Low risk |
| Stevens et al., 2024 | HPV | Adolescents | T: Providing reminders  C: Standard of Care | **Number of HPV shots at 12 months**  T vs. C: null  **Number of HPV shots at 18 months**  T vs. C: marginally positive | Total: 331  T: 166  C: 165 | 3-month intervention; follow-up to 18 months | High risk |
| Tang et al., 2019b | HIV/STI prevention | MSM | T: Crowdsourced educational materials;  C: Social marketing interventions. | **Self-reported condomless sex in the 3 weeks**  T vs. C: null  **Self-reported condomless sex in the 3 months**  T vs. C: null  **HIV testing in the 3 weeks**  T vs. C: null  **Syphilis testing in the 3 weeks**  T vs. C: null  T vs. C: null  **Syphilis testing in the 3 months**  T vs. C: null | Total: 1173  T: 586  C: 587 | 3 months | Low risk |
| Care engagement | | | | | | | |
| Hunter et al., 2023 | HIV | Adolescent girls and young women (AGYW) | T: Incentives (Voucher)  C: Standard of Care | **HIV self-test kits uptake**  T vs. C: positive  **Condom use：**  T vs. C: positive  **Linkage to HIV care**  T vs. C: null | Total: 20 drug shops  T: 10 drug shops  C: 10 drug shops | 4 months | Some concerns |
| Montoy et al., 2018 | HIV | Patients of a medical facility | T1: Incentives (Monetary)；  T2: Defaults (Active-choice, Opt-in, Opt-out)  C: Standard of Care | **HIV testing uptake**  T1-$1 vs. C1: null  T1-$5 vs. C1: positive  T1-$10 vs. C1: positive  T2: Active-choice vs. C2: positive  T2: Opt-out vs. C2: positive | Total: 6143  T1-$1: 768  T1-$5: 768  T1-$10: 768  C1: 768  T2: Active-choice: 1024  T2: Opt-out: 1024  C: 1023 | Single-visit intervention | Low risk |
| Montoy et al., 2016 | HIV | Patients of a medical facility | T1: Defaults (Active choice)  T2: Defaults (Opt-out)  C: Opt-in | **HIV testing uptake**  T1 vs. C: positive  T2 vs. C: positive | Total: 4800  T1: 1628  T2: 1565  C: 1607 | Single-visit intervention | High risk |
| Smith et al., 2021 | HIV | Male adults | T: Framing (Gain-framed)  C: Standard of Care | **Invited and tested for HIV**  T vs. C: positive  **Invited and came for HIV testing**  T vs. C: marginally positive  **Linked to ART：**  T vs. C: null | Total: 1048  T: 504  C: 544 | Single-visit intervention | Some concerns |
| Chamie et al., 2018 | HIV | Adult community members / general population | T1: Incentives (Lottery)  T2: Framing (Loss-framed)  C: Framing (Gain-framed) | **Tested for HIV**  T1 vs. C: marginally positive  T2 vs. C: null | Total: 2527  T1: 833  T2: 833  C: 861 | 13 days | Low risk |
| Chamie et al., 2021 | HIV | Adults | T1: Incentives (Monetary)  T2: Commitment (Deposit contracts)  C: Standard of Care | **HIV retest uptake at 3 & 6 months**  T1 vs. C: positive  T2 vs. C: null  T1 vs. T2: positive | Total:524  T1: 172  T2: 172  C: 180 | 6 months | Low risk |
| Ortiz et al., 2022 | HIV | Adults | T1: Soft-commitment  T2: Incentives (Monetary)  C: Information only | **HIV testing uptake**  T1 vs. C: positive  T2 vs. C: positive | Total: 425  T1: 138  T2: 177  C: 110 | Single-visit intervention | Some concerns |
| Macis et al., 2021 | HIV | Adults | T1: Soft-Commitment  T2: Immediate-Incentive (Monetary)  T3: Delayed-Incentive (Monetary)  C：Information alone | **HIV testing uptake**  T1 vs. C: null  T2 vs. C: positive  T3 vs. C: null | Total: 7720  T1: 3024  T2:805  T3: 1090  C: 2801 | Single-visit intervention | High risk |
| Brown et al., 2018 | HIV | Adults | T1: Planning prompts  T2: BE reminders  T3: Planning prompts + BE reminders  C: Standard reminders | **HIV testing kits returned(%)**  T1 vs. C: null  T2 vs. C: marginally positive  T3 vs. C: positive  T2 vs. T1: null T3 vs. T1: positive T3 vs. T2: null | Total: 8999  T1: 2275  T2: 2214  T3: 2267  C: 2243 | 3 months (short-term follow-up until kit return) | High risk |
| Kavanagh et al., 2020 | HIV | Adults | I: Planning prompts  C: Received a calendar | **HIV testing uptake**  T vs. C: null | Total: 2362  T: 1203  C: 1159 | 2 weeks (Single campaign period) | Low risk |
| Tang et al., 2016a | HIV | MSM | T: Crowdsourced educational materials  C: Health marketing | **Self-reported ﬁrst-time HIV testing**  T vs. C: null  **Secondary：**  **Cost per first-time HIV test**  T vs. C: null  **Cost per new HIV diagnosis**  T vs. C: null | Total: 624  T: 307  C: 317 | 3 weeks | Low risk |
| Lin et al., 2023 | HIV | MSM | T: Crowdsourced educational materials  C: Standard of Care | **HIV testing uptake at the 3 months**  T vs. C: null  **HIV testing uptake at the 6 months**  T vs. C: positive  **HIV testing uptake at the 9 months**  T vs. C: positive  **HIV testing uptake at the 12 months**  T vs. C: positive  **Testing for HIV in the previous 3 months**  T vs. C: null | Total: 697  T: 279  C: 418 | 12 months | Some concerns |
| Yan et al., 2024 | HIV | PLWH / index participants and their partners | T: Crowdsourced educational materials  C: Standard of Care | **Proportions of partners getting tested out of reported partners**  T vs. C: null  **Proportions of partners testing positive out of tested partners**  T vs. C: null | Total: 121  T: 81  C: 40 | 3 months | High risk |
| Mehta et al., 2021 | HCV | Adults in healthcare setting | T1: Defaults + Letter  T2: Norms  C1: Letter only  C2: Standard of Care | **HCV screening at 4 months**  T1 vs. C1：positive  T2 vs. C2：marginally positive  **HCV screening at 12 months**  T1 vs. C1：positive  T2 vs. C2：null | Total1: 1642 (T1+C1)  Total2: 19,661 (T2+C2)  T1: 829  T2: 9833  C1: 813  C2: 9828 | 12 months | Low risk |
| Niza et al., 2014 | Chlamydia | Young adults | T1: Incentives (voucher) -Framing (Gain-framed)  T2: Incentives (voucher) -Framing (loss-framed)  T3: Incentives(Lottery)- Framing (Gain-framed)  T4: Incentives(Lottery) -Framing (Loss-framed)  C: No incentives | **Chlamydia screening kits returned (%)**  T1+T2+ T3+T4 vs. C: positive  T3+T4 vs. T1+T2: positive  T1+T3 vs. T2+T4: null | Total: 1060  T/C: Not reported (cluster RCT; arm-level n not reported) | Single screening round (Short-term follow-up until kit return) | Some concerns |
| Fitzpatrick et al., 2019 | HBV/HCV | MSM | T: Crowdsourced educational materials  C: Standard of Care | **Confirmed HBV and HCV test uptake at 4 weeks**  T vs. C: null  **Self-reported HBV and HCV test uptake at 4 weeks**  T vs. C: null  **HBV vaccination uptake at 4 weeks**  T vs. C: null  **HIV test uptake at 4 weeks**  T vs. C: null  **Chlamydia test uptake at 4 weeks**  T vs. C: null  **Gonorrhea test uptake at 4 weeks**  T vs. C: null  **Syphilis test uptake at 4 weeks**  T vs. C: null  **Visit with a physician after hepatitis test**  T vs. C: null | Total: 556  T: 280  C: 276 | 4 weeks | High risk |
| Wong et al., 2022 | HBV/HCV | MSM | T: Crowdsourced educational materials  C: Standard of Care | **Primary：**  **Confirmed HBV testing at 4 weeks**  T vs. C: null  **Confirmed HCV testing at 4 weeks**  T vs. C: null  **Confirmed both HBV and HCV test at 4 weeks**  T vs. C: null  **Confirmed HBV positives at 4 weeks**  T vs. C: null  **Confirmed HCV positive at 4 weeks**  T vs. C: null  **Sought medical care after receiving HBV results (self-report) at 4 weeks**  T vs. C: null  **Sought medical care after receiving HCV results (self-report) at 4 weeks**  T vs. C: null  **Self-reported HBV vaccination at 4 weeks**  T vs. C: null | Total: 750  T: 376  C: 374 | 4 weeks | High risk |
| Yang et al., 2020 | Chlamydia/Gonorrhoea | MSM | T1: Pay-it-forward  T2: Pay-what-you-want  C: Standard of Care | **Dual chlamydia and gonorrhea testing uptake at the intervention visit**  T1 vs. C: positive  T2 vs. C: positive  **The economic cost per person tested**  T1 vs. C: ICER = US$12·96  T2 vs. C: ICER = US$12·68  T1 vs. T2: ICER = US$14·27 | Total: 301  T1: 101  T2: 100  C: 100 | Single-visit intervention | Some concerns |
| Tang et al., 2023 | Chlamydia/Gonorrhoea | Female sex workers | T: Pay-it-forward  C: Standard of Care | **Dual Chlamydia and Gonorrhea testing uptake at the intervention visit**  T vs. C: positive  **The economic cost per person tested**  T VS. C: ICER = US $ -2·79 | Total: 480  T: 240  C: 240 | Single-visit intervention | High risk |
| Zhang et al., 2023 | HBV/HCV | MSM | T: Pay-it-forward  C: Standard of Care | **The proportion of dual HBV and HCV testing uptake**  T vs. C: positive  **The economic cost per person tested**  T vs. C: ICER (Not reported) Dominated. | Total: 322  T: 160  C: 162 | Single-visit intervention | Low risk |
| Bokolo et al., 2024 | HIV | Adults living with HIV | T1: Framing (Gain-framed)  T2: Incentive (Care voucher)  C: Standard of Care | **HIV clinic visits within 4 months**  T1 vs. C: null  T2 vs. C: null | Total: 11,435  T1: 3804  T2: 3829  C: 3802 | 4 months | Some concerns |
| Maughan-Brown et al., 2018 | HIV | Adults newly diagnosed with HIV | T: Incentive (Voucher)  C: Standard of Care | **Linkage to HIV care within 3 months**  T vs. C: null  **ART initiation within 3 months**  T vs. C: null | Total: 86  T: 46  C: 40 | 3 months | High risk |
| Barnabas et al., 2020 | HIV | Adults newly diagnosed with HIV | T: Incentives (Lottery)  C: Standard of Care | **Linkage to the ART clinic**  T vs. C: null  **Initiated ART**  T vs. C: null  **HIV viral suppression (Viral load <20 copies/mL)**  T vs. C: null | Total: 131  T: 56  C: 75 | 6 months | Low risk |
| Treatment adherence | | | | | | | |
| Yotebieng et al., 2016 | HIV | PLWH(Pregnant women living with HIV) | T: Incentives(Monetary)  C: Standard of Care | **Retention in HIV care at 6 weeks postpartum**  T vs. C: positive  **Attended each clinic visit and received services**  T vs. C: positive | Total: 433  T: 216  C: 217 | ≤32 weeks pregnant to 6 weeks postpartum | Low risk |
| Fahey et al., 2020 | HIV | PLWH | T1: Incentives (Monetary)- $4·50  T2: Incentives (Monetary)- $10  C: Standard of Care | **Retained in HIV care and viral suppression (<1000 copies per mL) at 6 months**  T1 vs. C: positive T2 vs. C: positive  **Retention on ART at 6 months**  T1 vs. C: null  T2 vs. C: positive  **HIV viral suppression (<1000 copies per mL) at 6 months**  T1 vs. C: marginally positive  T2 vs. C: positive | Total: 530  T1: 172  T2: 174  C: 184 | 6 months | Low risk |
| Silverman et al., 2019 | HIV | PLWH | T: Incentives (Monetary)  C: Standard of Care | **HIV viral suppression**  T vs. C: positive  **Self-reported ART adherence (> 90%)**  T vs. C: positive | Total: 102  T: 52  C: 50 | 12 months | Some concerns |
| Alsan et al., 2017 | HIV | PLWH | T1:Incentives(Monetary)-provider visit incentive  T2: Incentive Choice - Monetary or commitment contract  C: Standard of Care | **HIV viral suppression at 5th visit**  T2 vs. T1: null  T2 vs. C: null  **HIV viral suppression at 6th visit**  T2 vs. T1: null  T2 vs. C: positive | Total: 110  T1: 21  T2: 19  C: 70 | 6-months | Some concerns |
| Thirumurthy et al., 2019 | HIV | PLWH | T: Incentives (Monetary)  C: Standard of Care | **HIV viral suppression at 6 months**  T vs. C: null | Total: 400  T: 203  C: 197 | 6 months | High risk |
| Linnemayr et al., 2024 (a) | HIV | PLWH | T1: Messages(salience)  T2: Incentives (monetary)  C: Standard of Care | **Mean adherence to ART at 1-3months (Intervention period)**  T1 vs. C: null  T2 vs. C: positive  **Mean adherence to ART at 4-6 months**  T1 vs. C: null  T2 vs. C: positive  **Mean adherence to ART at 7-9 months**  T1 vs. C: null  T2 vs. C: marginally positive | Total:155  T1: 49  T2: 57  C: 49 | 9 months | High risk |
| Linnemayr et al., 2024 (b) | HIV | PLWH | T1: Clinic-linked Incentives(Lottery)  T2: Adherence-linked Incentives(Lottery)  C: Standard of Care | **Mean adherence to ART**  T1+T2 vs. C: null  T1 vs. C: null  T2 vs. C: marginally positive  **HIV viral suppression**  T1+T2 vs. C: null  T1 vs. C: null  T2 vs. C: null | Total = 329  T1 = 109  T2 = 111  C = 109 | 12 months | Some concerns |
| Linnemayr et al., 2017 | HIV | PLWH | T1: Clinic-linked incentives (Lottery)  T2: 90% adherence-linked incentive(Lottery)  C：Standard of Care | **Mean ART adherence over 9 months**  T1+T2: vs. C: null  T1 vs. C: marginally positive  T2 vs. C: null  **Mean ART adherence (> 90%) over 9 months**  T1+T2: vs. C: positive  T1 vs. C: marginally positive  T2 vs. C: positive | Total: 146  T1: 46  T2: 52  C: 48 | 9 months | Some concerns |
| Stecher et al., 2021 | HIV | PLWH | T1: Clinic-linked incentives(Lottery)  T2: Adherence-linked incentives(Lottery)  C: Standard of Care | **Mean ART Adherence over 20 Months**  T1 vs. C：null  T2 vs. Cl：null | Total: 208  T1: 70  T2: 69  C: 69 | 20 months | High risk |
| Bien-Gund et al., 2021 | HIV | PLWH | T: Incentives(Lottery)；  C: Standard of Care | **HIV viral suppression (viral load<400 copies/ml)**  T vs. C: null | Total: 29  T: 15  C: 14 | 3 months | Some concerns |
| MacCarthy et al., 2020 | HIV | PLWH | T1: Received own adherence level texts  T2: Norms (Received own & peers' adherence level texts·)  C: Standard of Care | **Mean ART adherence over the 9 months**  T1 vs. C: null  T2 vs. C: null | Total: 155  T1: 52  T2: 54  C: 49 | 9 months | Some concerns |

**Abbreviations:** ART, antiretroviral therapy; HBV, hepatitis B virus; HCV, hepatitis C virus; HIV, human immunodeficiency virus; HPV, human papillomavirus; MMC, medical male circumcision; MSM, men who have sex with men; PLWH, people living with HIV; RoB, risk of bias; STBBI, sexually transmitted and blood-borne infection.

^a^ Marginally positive: P < 0.1; positive: P < 0.05, P < 0.01, or P < 0.001.

^b^ Overall RoB judgement was assessed using Cochrane RoB 2; for cluster randomised trials, the randomisation domain was derived by combining the judgements for randomisation and the timing of participant identification/recruitment.

^c^ Monetary incentives were used to promote safer sexual practices, reducing the prevalence of four STIs: Chlamydia trachomatis, Neisseria gonorrhoeae, Trichomonas vaginalis, and Mycoplasma genitalium.

^d^ “Null” indicates no statistically significant difference between the intervention and comparison groups.

## Reference

1. Hanoch Y, Barnes A, Rice T. Behavioral economics and healthy behaviors: Key concepts and current research. 2017

2. Reina Ortiz M, Grunauer M, Gutierrez E, et al. Financial Incentives, Not Behavioral Nudges, Led to Optimized HIV Testing among Pregnant Women in a High-Burden Urban Population in Ecuador. *American journal of tropical medicine and hygiene* 2022;106(6):1703‐10. doi: doi:10.4269/ajtmh.21-0591

3. Montoy JCC, Dow WH, Kaplan BC. Cash incentives versus defaults for HIV testing: a randomized clinical trial. *PLoS One* 2018;13(7):e0199833.

4. De Walque D, Dow WH, Nathan R, et al. Incentivising safe sex: a randomised trial of conditional cash transfers for HIV and sexually transmitted infection prevention in rural Tanzania. *BMJ Open* 2012;2(1):e000747.

5. Chamie G, Kwarisiima D, Ndyabakira A, et al. Financial incentives and deposit contracts to promote HIV retesting in Uganda: A randomized trial. *PLoS Med* 2021;18(5):e1003630. doi: 10.1371/journal.pmed.1003630 [published Online First: 2021/05/05]

6. Macis M, Grunauer M, Gutierrez E, et al. Using Incentives and Nudging to Improve Non-Targeted HIV Testing in Ecuador: a Randomized Trial. *AIDS and behavior* 2021;25(8):2542‐50. doi: doi:10.1007/s10461-021-03215-x

7. Alsan M, Beshears J, Armstrong WS, et al. A commitment contract to achieve virologic suppression in poorly adherent patients with HIV/AIDS. *AIDS (London, England)* 2017;31(12):1765‐69. doi: doi:10.1097/QAD.0000000000001543

8. Fahey CA, Njau PF, Katabaro E, et al. Financial incentives to promote retention in care and viral suppression in adults with HIV initiating antiretroviral therapy in Tanzania: a three-arm randomised controlled trial. *The Lancet HIV* 2020;7(11):e762-e71.

9. Yotebieng M, Thirumurthy H, Moracco KE, et al. Conditional cash transfers and uptake of and retention in prevention of mother-to-child HIV transmission care: a randomised controlled trial. *The lancet HIV* 2016;3(2):e85-e93.

10. Silverman K, Holtyn AF, Rodewald AM, et al. Incentives for viral suppression in people living with HIV: a randomized clinical trial. *AIDS and Behavior* 2019;23:2337-46.

11. Thirumurthy H, Ndyabakira A, Marson K, et al. Financial incentives for achieving and maintaining viral suppression among HIV-positive adults in Uganda: a randomised controlled trial. *Lancet HIV* 2019;6(3):e155-e63. doi: doi:10.1016/s2352-3018(18)30330-8 10.1016/S2352-3018(18)30330-8. Epub 2019 Jan 16.

12. Linnemayr S, Odiit M, Mukasa B, et al. INcentives and ReMINDers to Improve Long-Term Medication Adherence (INMIND): impact of a pilot randomized controlled trial in a large HIV clinic in Uganda. *J Int AIDS Soc* 2024;27(6):e26306. doi: 10.1002/jia2.26306 [published Online First: 2024/06/26]

13. Thaler RH, Sunstein CR. Nudge: Improving decisions about health, wealth, and happiness: Penguin 2009.

14. Thirumurthy H, Masters SH, Rao S, et al. The Effects of Providing Fixed Compensation and Lottery-Based Rewards on Uptake of Medical Male Circumcision in Kenya: a Randomized Trial. *Journal of acquired immune deficiency syndromes (1999)* 2016;72:S299‐305. doi: doi:10.1097/QAI.0000000000001045

15. Chamie G, Schaffer EM, Ndyabakira A, et al. Comparative effectiveness of novel nonmonetary incentives to promote HIV testing. *Aids* 2018;32(11):1443-51. doi: 10.1097/qad.0000000000001833 [published Online First: 2018/04/24]

16. Niza C, Rudisill C, Dolan P. Vouchers versus Lotteries: What Works Best in Promoting Chlamydia Screening? A Cluster Randomized Controlled Trial. *Appl Econ Perspect Policy* 2014;36(1):109-24. doi: doi:10.1093/aepp/ppt033

17. Galárraga O, Harries J, Maughan-Brown B, et al. The Empower Nudge lottery to increase dual protection use: a proof-of-concept randomised pilot trial in South Africa. *Reproductive health matters* 2018;26(52):1510701. doi: doi:10.1080/09688080.2018.1510701

18. Bien-Gund CH, Ho JI, Bair EF, et al. Brief report: financial incentives and real-time adherence monitoring to promote daily adherence to HIV treatment and viral suppression among people living with HIV: a pilot study. *JAIDS Journal of Acquired Immune Deficiency Syndromes* 2021;87(1):688-92.

19. Linnemayr S, Wagner Z, Saya UY, et al. Behavioral economic incentives to support HIV care: Results from a randomized controlled trial in Uganda. *J Acquir Immune Defic Syndr* 2024 doi: doi:10.1097/qai.0000000000003420 10.1097/QAI.0000000000003420.

20. Linnemayr S, Stecher C, Mukasa B. Behavioral economic incentives to improve adherence to antiretroviral medication. *AIDS (London, England)* 2017;31(5):719‐26. doi: doi:10.1097/QAD.0000000000001387

21. Stecher C, Mukasa B, Linnemayr S. Uncovering a behavioral strategy for establishing new habits: evidence from incentives for medication adherence in Uganda. *Journal of health economics* 2021;77:102443. doi: 10.1016/j.jhealeco.2021.102443

22. Barnabas RV, van Heerden A, McConnell M, et al. Lottery incentives have short-term impact on ART initiation among men: results from a randomized pilot study. *Journal of the International AIDS Society* 2020;23:e25519. doi: doi:10.1002/jia2.25519

23. Bokolo S, Mabaso S, Kruger W, et al. Applying behavioural economics principles to increase demand for free HIV testing services at private doctor-led clinics in Johannesburg, South Africa: A randomised controlled trial. *PLOS Global Public Health* 2024;4(8):e0003465.

24. Thirumurthy H, Masters SH, Rao S, et al. Effect of providing conditional economic compensation on uptake of voluntary medical male circumcision in Kenya: a randomized clinical trial. *Jama* 2014;312(7):703-11.

25. Hunter LA, Rao A, Napierala S, et al. Reaching Adolescent Girls and Young Women With HIV Self-Testing and Contraception at Girl-Friendly Drug Shops: A Randomized Trial in Tanzania. *J Adolesc Health* 2023;72(1):64-72. doi: 10.1016/j.jadohealth.2022.08.013 [published Online First: 2022/10/15]

26. Maughan-Brown B, Smith P, Kuo C, et al. A conditional economic incentive fails to improve linkage to care and antiretroviral therapy initiation among HIV-positive adults in Cape Town, South Africa. *AIDS patient care and STDs* 2018;32(2):70-78.

27. Montoy JCC, Dow WH, Kaplan BC. Patient choice in opt-in, active choice, and opt-out HIV screening: randomized clinical trial. *bmj* 2016;352

28. Mehta SJ, Day SC, Norris AH, et al. Behavioral interventions to improve population health outreach for hepatitis C screening: randomized clinical trial. *BMJ (Clinical research ed)* 2021;373:n1022. doi: doi:10.1136/bmj.n1022

29. Smith P, Buttenheim A, Schmucker L, et al. Undetectable= untransmittable (U= U) messaging increases uptake of HIV testing among men: results from a pilot cluster randomized trial. *AIDS and Behavior* 2021;25(10):3128-36.

30. Roberto CA, Kawachi I. Behavioral economics and public health: Oxford University Press 2015.

31. Kahneman D. Maps of Bounded Rationality: Psychology for Behavioral Economics. *American Economic Review* 2003;93(5):1449–75. doi: 10.1257/000282803322655392

32. MacCarthy S, Wagner Z, Mendoza-Graf A, et al. A randomized controlled trial study of the acceptability, feasibility, and preliminary impact of SITA (SMS as an Incentive To Adhere): a mobile technology-based intervention informed by behavioral economics to improve ART adherence among youth in Uganda. *BMC infectious diseases* 2020;20(1):173. doi: doi:10.1186/s12879-020-4896-0

33. Brown LJ, Tan KS, Guerra LE, et al. Using behavioural insights to increase HIV self-sampling kit returns: a randomized controlled text message trial to improve England's HIV self-sampling service. *HIV medicine* 2018;19(9):585‐96. doi: doi:10.1111/hiv.12634

34. Kavanagh NM, Schaffer EM, Ndyabakira A, et al. Planning prompts to promote uptake of HIV services among men: a randomised trial in rural Uganda. *BMJ global health* 2020;5(11) doi: doi:10.1136/bmjgh-2020-003390

35. Koch AK, Mønster D, Nafziger J. Spillover effects of reminder nudges in complex environments. *Proceedings of the National Academy of Sciences* 2024;121(17):e2322549121.

36. Stevens J, Rausch J, Osuagwu N, et al. Efficacy of Behavioral Economic Nudges to Assist Teen Mothers: the Healthy Adolescent Transitions Randomized Controlled Trial. *Prevention science* 2024 doi: doi:10.1007/s11121-024-01660-3

37. Dolan P, Hallsworth M, Halpern D, et al. Influencing behaviour: The mindspace way. *Journal of economic psychology* 2012;33(1):264-77.

38. Li KT, Tang W, Wu D, et al. Pay-it-forward strategy to enhance uptake of dual gonorrhea and chlamydia testing among men who have sex with men in China: a pragmatic, quasi-experimental study. *Lancet Infect Dis* 2019;19(1):76-82. doi: 10.1016/s1473-3099(18)30556-5 [published Online First: 2018/12/28]

39. Gray K, Ward AF, Norton MI. Paying it forward: generalized reciprocity and the limits of generosity. *J Exp Psychol Gen* 2014;143(1):247-54. doi: 10.1037/a0031047 [published Online First: 2012/12/19]

40. Yang F, Zhang TP, Tang W, et al. Pay-it-forward gonorrhoea and chlamydia testing among men who have sex with men in China: a randomised controlled trial. *Lancet Infect Dis* 2020;20(8):976-82. doi: 10.1016/s1473-3099(20)30172-9 [published Online First: 2020/06/13]

41. Tang W, Xie Y, Xiong M, et al. A Pay-It-Forward Approach to Improve Chlamydia and Gonorrhea Testing Uptake Among Female Sex Workers in China: Venue-Based Superiority Cluster Randomized Controlled Trial. *JMIR Public Health Surveill* 2023;9:e43772. doi: 10.2196/43772 [published Online First: 2023/03/03]

42. Zhang Y, Li JJ, Xie YW, et al. Pay-it-forward incentives for hepatitis virus testing in men who have sex with men: a cluster randomized trial. *Nat Med* 2023;29(9):2241-+. doi: 10.1038/s41591-023-02519-w

43. Bender E. Challenges: Crowdsourced solutions. *Nature* 2016;533(7602):S62-4. doi: 10.1038/533S62a [published Online First: 2016/05/12]

44. Fitzpatrick T, Tang W, Mollan K, et al. A crowdsourced intervention to promote hepatitis B and C testing among men who have sex with men in China: A nationwide online randomized controlled trial. *EClinicalMedicine* 2019;16:64-73. doi: 10.1016/j.eclinm.2019.10.007 [published Online First: 2019/12/14]

45. Wong WCW, Marley G, Li J, et al. A randomized controlled trial enhancing viral hepatitis testing in primary care via digital crowdsourced intervention. *NPJ Digit Med* 2022;5(1):95. doi: 10.1038/s41746-022-00645-2 [published Online First: 2022/07/20]

46. Tang W, Mao J, Liu C, et al. Reimagining Health Communication: A Noninferiority Randomized Controlled Trial of Crowdsourced Intervention in China. *Sex Transm Dis* 2019;46(3):172-78. doi: 10.1097/olq.0000000000000930 [published Online First: 2019/02/12]

47. Yan X, Tucker JD, Miller WC, et al. Crowdsourced Partner Services Among Men Who Have Sex With Men Living With HIV: A Pilot Randomized Controlled Trial in China. *Sex Transm Dis* 2024;51(10):673-80. doi: 10.1097/olq.0000000000001989 [published Online First: 2024/05/01]

48. Tang W, Han L, Best J, et al. Crowdsourcing HIV Test Promotion Videos: A Noninferiority Randomized Controlled Trial in China. *Clin Infect Dis* 2016;62(11):1436-42. doi: 10.1093/cid/ciw171 [published Online First: 2016/05/01]

49. Lin Y, Ren C, Liao M, et al. Digital, Crowdsourced, Multilevel Intervention to Promote HIV Testing Among Men Who Have Sex With Men: Cluster Randomized Controlled Trial. *J Med Internet Res* 2023;25:e46890. doi: 10.2196/46890 [published Online First: 2023/10/30]
